# Epithelial and interstitial Notch1 activity contributes to the myofibroblastic phenotype and fibrosis

**DOI:** 10.1186/s12964-019-0455-y

**Published:** 2019-11-12

**Authors:** Weilong Hong, Ge Zhang, Hong Lu, Yangyang Guo, Shizhang Zheng, Hengyue Zhu, Yanyi Xiao, Akuetteh Percy David Papa, Cunzao Wu, Linxiao Sun, Bicheng Chen, Yongheng Bai

**Affiliations:** 1 Key Laboratory of Diagnosis and Treatment of Severe Hepato-Pancreatic Diseases of Zhejiang Province, The First Affiliated Hospital of Wenzhou Medical University, Wenzhou, 325000 China; 2 Department of Orthopedics, People’s Hospital of Luzhou City, Luzhou, 646000 China; 3 Department of Laboratory Medicine, The First Affiliated Hospital of Wenzhou Medical University, Wenzhou, 325000 China; 4 Department of Transplantation, The First Affiliated Hospital of Wenzhou Medical University, Wenzhou, 325000 China; 5 Institute of Kidney Health, Center for Health Assessment, Wenzhou Medical University, Wenzhou, 325000 China

**Keywords:** Notch1 signalling, Myofibroblastic phenotype, Epithelial-mesenchymal transition (EMT), Fibroblast-myofibroblast differentiation (FMD), Renal fibrosis

## Abstract

**Background:**

Notch1 signalling is a stem-cell-related pathway that is essential for embryonic development, tissue regeneration and organogenesis. However, the role of Notch1 in the formation of myofibroblasts and fibrosis in kidneys following injury remains unknown.

**Methods:**

The activity of Notch1 signalling was evaluated in fibrotic kidneys in CKD patients and in ureteral obstructive models in vivo and in cultured fibroblasts and TECs in vitro. In addition, the crosstalk of Notch1 with TGF-β1/Smad2/3 signalling was also investigated.

**Results:**

Notch1 activity was elevated in fibrotic kidneys of rat models and patients with chronic kidney disease (CKD). Further study revealed that epithelial and interstitial Notch1 activity correlated with an α-SMA-positive myofibroblastic phenotype. In vitro, injury stimulated epithelial Notch1 activation and epithelial-mesenchymal transition (EMT), resulting in matrix deposition in tubular epithelial cells (TECs). Additionally, interstitial Notch1 activation in association with fibroblast-myofibroblast differentiation (FMD) in fibroblasts mediated a myofibroblastic phenotype. These TGF-β1/Smad2/3-dependent phenotypic transitions were abolished by Notch1 knockdown or a specific antagonist, DAPT, and were exacerbated by Notch1 overexpression or an activator Jagged-1-Fc chimaera protein. Interestingly, as a major driving force behind the EMT and FMD, TGF-β1, also induced epithelial and interstitial Notch1 activity, indicating that TGF-β1 may engage in crosstalk with Notch1 signalling to trigger fibrogenesis.

**Conclusion:**

These findings suggest that epithelial and interstitial Notch1 activation in kidneys following injury contributes to the myofibroblastic phenotype and fibrosis through the EMT in TECs and to the FMD in fibroblasts by targeting downstream TGF-β1/Smad2/3 signalling.

## Background

Renal fibrosis is a global health issue that causes morbidity and mortality and has no currently available treatment. Renal fibrosis is a dynamic convergence process, characterized by excessive accumulation of extracellular matrix (ECM), including collagens I and III, in the interstitium of the kidney [1, 2]. With crucial roles in fibrosis, alpha-smooth muscle actin (α-SMA)-positive myofibroblasts are responsible for the synthesis of ECM components [3]. However, the origin of myofibroblasts is still a matter of debate [4]. Evidence indicates that myofibroblasts are derived from tubular epithelial cells (TECs) through the epithelial-mesenchymal transition (EMT) programme [5–7]. Recent investigations have also highlighted that a large proportion of myofibroblasts originate from local resident fibroblasts through fibroblast-myofibroblast differentiation (FMD) [6]. Thus, novel therapeutic strategies that halt or perhaps reverse the FMD in fibroblasts and the EMT in TECs to attenuate the myofibroblastic phenotype and fibrosis are promising.

As adaptive responses after persistent injury, the EMT or FMD may be an integral part of fibrosis [8–10]. During the EMT, TECs lose epithelial phenotypes including E-cadherin, and acquire new characteristic features of mesenchymal proteins, such as α-SMA [8]. Similarly, in FMD, fibroblasts undergo remodelling behaviour and matrix structural changes [9, 10]. The major driving force behind EMT and FMD during the fibrogenic phase of fibrosis appears to be various growth factors, such as transforming growth factor-β1 (TGF-β1). Amelioration of EMT with the treatment of TGF-β1 signalling antagonists bone morphogenetic protein-7 (BMP-7) or Smad7 significantly alleviates fibrotic lesions [11, 12]. Emerging evidence has shown that enhanced TGF-β1 levels may be associated with aberrant activation of Notch1 signalling [13, 14]. As a stem-cell-related signalling process, Notch1 signalling plays crucial roles in tissue regeneration, organogenesis and embryonic development [15, 16]. Over-activation of Notch1 signalling results in a series of pathological consequences, including different types of tumours [17, 18]. In addition to these effects, activated Notch1 signalling is also reported to contribute to fibrosis in many tissues, including the kidney [19]. In injured cells, Notch1 signalling is activated through the binding of the ligand Jagged to its membrane receptor, Notch1 protein. NICD is an active intracellular domain of Notch1 protein and translocates to the nucleus to stimulate a signalling cascade [20]. As a result, activated Notch1 signalling inhibits apoptosis and promotes cellular proliferation and differentiation through regulating the expression levels of cell-cycle-related factors, such as cyclin D1, CDK2 and p21 [21]. Recent studies have shown that Notch1 signalling activation contributes to the development of albuminuria, glomerulosclerosis and kidney dysfunction [22] and regulates interstitial fibrosis progress in the kidneys of mice and humans [19]. Blockade of Notch1 signalling ameliorates diabetic kidney disease, nephrotic syndrome and fibrosis [19, 23]. Thus, Notch1 signalling plays a crucial role in the development of renal fibrosis [23]. However, the underlying molecular mechanisms by which Notch1 signalling drives the fibrotic response have not been fully elucidated.

To explore the functional role of the Notch1 signalling pathway in fibrosis following injury, we examined the activity of Notch1 signalling in fibrotic kidneys in CKD patients and ureteral obstructive models in vivo and in cultured fibroblasts and TECs in vitro. In addition, the crosstalk of Notch1 with TGF-β1/Smad2/3 signalling was also investigated. Our findings suggest that the activation of epithelial and interstitial Notch1 signalling following injury contributes to the myofibroblastic phenotype and renal fibrosis through the EMT in TECs and to the FMD in fibroblasts by targeting downstream TGF-β1/Smad2/3 signalling. Pharmacologic inhibition of Notch1 signalling may have therapeutic potential for fibrotic kidney diseases.

## Materials and methods

### Antibodies and reagents

Antibodies and reagents were obtained as follows: anti-Notch1 (western blot (WB), 1:800; immunohistochemical (IHC) or immunofluorescence (IF) staining, 1:400), anti-E-cadherin (WB and IF, 1:400), anti-N-cadherin (WB, 1:400), anti-α-SMA (WB, 1:800, IHC and IF, 1:400), and anti-Ki67 (IHC and IF, 1:400) antibodies from Cell Signalling Technology (CST, Beverly, MA, USA); anti-NICD (WB, 1:400, IHC and IF, 1:200), anti-Histone H3 (WB, 1:800), anti-c-Myc (WB, 1:800), and anti-vimentin (WB, 1:800; IHC, 1:400) antibodies from Abcam Company (Cambridge, MA, USA); anti-Jagged1 (WB, 1:800) antibody from Santa Cruz Biotechnology; anti-Smad2/Smad3 (phospho T8) (WB, 1:800) and anti-TGF-β1R (WB, 1:800) antibodies from MDL Biotechnology (Beijing, China); anti-GAPDH (WB, 1:8000), anti-Smad2/3 (WB and IF, 1:1000), anti-p-Smad2 (WB, 1:1000), anti-p-Smad3 (WB, 1:1000), anti-c-Myc (WB, 1:800); anti-collagen I (WB, 1:800; IF, 1:200), and anti-collagen III (WB, 1:800, IHC and IF, 1:200) antibodies from Biogot Technology (Shanghai, China); and anti-TGF-β1 (WB, 1:800) and anti-β-actin (WB, 1:8000) antibodies from Proteintech Biotechnology (Wuhan, China).

### Human kidney tissue samples

The tissues studied were drawn from banks of renal samples snap-frozen immediately after they were obtained and were maintained at The First Affiliated Hospital of Wenzhou Medical University, Wenzhou, China. The fibrotic kidney tissues were obtained from 15 patients with chronic kidney diseases. Normal non-fibrotic control kidney samples were from 18 patients with minimal change nephropathy. Tissues were studied in accordance with institutional guidelines for research involving human subjects, and this study was approved by the Ethics Committee of The First Affiliated Hospital of Wenzhou Medical University.

### Cell lines and drug treatment

Rat kidney fibroblast (NRK-49F) and tubular epithelium (NRK-52E) cell lines were obtained from the Cell Bank of the Chinese Academy of Sciences (Shanghai, China). Cells were maintained in Dulbecco’s Modified Eagle Medium (DMEM, HyClone, Logan, UT, USA) supplemented with 5% foetal bovine serum (FBS, Gibco, Grand Island, NY, USA), 100 U/ml penicillin and 100 μg/ml streptomycin (Gibco). Cells were seeded on six-well culture plates to approximately 70% confluence in complete medium containing 5% FBS for 24 h and were then changed to serum-free medium for 24 h before treatment with 5 ng/ml TGF-β1 protein (PeproTech, Rocky Hill, NJ, USA), 1 μg/ml recombinant Jagged 1 Fc Chimera protein (Jag1-Fc, R&D Systems, Shanghai, China), or 10 ng/ml AA (Sigma-Aldrich, St. Louis, MO, USA) with or without 1 or 10 μmol/L DAPT (Sigma-Aldrich).

### Rats and animal experiments

Six- to 8-week-old male Sprague-Dawley rats obtained from the Experimental Animal Center of Wenzhou Medical University (Wenzhou, China) were used in this study. The Institutional Animal Care and Use Committee of Wenzhou Medical University (China) approved all animal study protocols. Rats were housed under a controlled temperature (22–25 °C), humidity (40–60%) and light environment (12-h dark/light) and were fed with a standard rat chow and water, except for 1 day fasting before the operation. Weight-matched rats were randomly assigned to four groups, as follows: i) Sham-operation group (Sham, *n* = 8); ii) UUO group (vehicle, n = 8); iii) DAPT (100 mg/kg)-treated Sham group (n = 8); and iv) DAPT (100 mg/kg)-treated UUO group (n = 8). All UUO surgery was performed as previously described after the rats were anaesthetized by intraperitoneal injection with 2% Nembutal (0.3 ml/100 g). At the same time and every 24 h after the establishment of the UUO, the rats of each group were intraperitoneally injected with DAPT until the kidneys were excised on day 7 after obstruction operation. UUO surgery was performed as previously described [24].

### Histopathological examination

Kidney tissues were fixed in 10% neutral buffered formalin and embedded in paraffin. Four-micron sections were cut for haematoxylin and eosin (HE, Yuanye Biotechnology, Shanghai, China) and Masson’s trichrome (Yuanye Biotechnology) staining according to the manufacturer’s protocol. Slides were examined and pictures were taken using a DM4000B LED Microscope System (Leica Microsystems, Germany) and a DFC 420C 5 M Digital Microscope Camera (Leica Microsystems). Tubulointerstitial damage and the degree of interstitial collagen deposition were graded as described previously [25].

### Immunohistochemical staining

Formalin-fixed, paraffin-embedded kidney sections were dewaxed through xylene and hydrated through graded ethanol (100, 95, 85, and 75%) and distilled water. Endogenous peroxidase was blocked with 3% hydrogen peroxide. Antigen retrieval was performed by heating in 0.1% sodium citrate buffer (pH 6.0). Immunohistochemical studies were semiquantitatively or quantitatively assessed by two independent investigators in a blinded manner.

### Microarray experiment and GO classification

Total RNA was prepared from whole kidneys using TRIzol (Invitrogen). Gene expression studies were performed using the OneArray Plus microarray kit (Genechem Biotechnology, Shanghai, China) according to the manufacturer’s instructions. The raw data files were imported into the Rosetta Resolver® System (Rosetta Biosoftware, Seattle, WA, USA) to analyse the gene expression data after hybridization and scanning. The error model was used to evaluate the credibility of the probe signal, and median scaling was used to normalize the database and expression levels. The normalizations standardized the data to facilitate the identification of genuine differences in gene expression. The log2 (ratios) and *p*-values of the differentially expressed genes were calculated. All significant gene entries were subjected to PCA (Principal Component Analysis) and clustering analysis using the R Statistical Package (version 3.0.3).

### RTCA analysis for cell proliferation

In studies, 10% FBS served as the stimulator, and the cells were cultured in serum-free medium for 24 h before experimentation. Real-time monitoring of cell proliferation was performed using an xCELLigence MP system (ACEA Biosciences, San Diego, USA). E-plate 96, used with the xCELLigence system, is a single-use 96-well cell culture plate with its bottom surfaces covered with microelectrode sensors (0.2 cm^2^ well surface area; 243 ± 5 μl maximum volumes). Real-time changes in electrical impedance were measured using gold microelectrodes and are expressed as “cell index” defined as (Rn-Rb)/15, where Rb is the background impedance and Rn is the impedance of the well with cells.

Before seeding cells into E-plate 96, the background impedance was measured after the addition of 100 μl of medium and a 30 min-incubation period at room temperature. Cell density was determined using a haemocytometer after methylene blue staining. Following the seeding of the appropriate number of cells into the wells, the plate was incubated at room temperature for 30 min to allow cell settling. Cellular proliferation was monitored every 30 min for over 24 h.

### Immunofluorescence staining

NRK-52E cells or NRK-49F cells were cultured in six-well plates containing glass slides and were then washed with PBS and fixed with 4% paraformaldehyde (Sigma-Aldrich) at 4 °C for 30 min. After permeabilization with 0.1% Triton X-100 for 10 min, the specimens were washed with PBS and then blocked with 10% FBS to eliminate the nonspecific fluorescence. Immunofluorescence staining was performed using anti-Ki67, α-SMA, NICD, Notch1, Jagged1, and collagen III as the primary antibodies, and the cell preparations were incubated with Alexa Fluor 488 (green)- or 594 (red) (Proteintech)-labelled secondary antibodies. Immunocytochemical samples were semi-quantitatively or quantitatively assessed by two independent investigators in a blinded manner.

### RNA isolation and quantitative real-time PCR

Total RNA was extracted from rat kidneys using TRIzol reagent (Invitrogen) and reverse-transcribed to cDNA templates using a ReverTra Ace qPCR RT Kit (Toyobo, Japan). Quantitative real-time PCR (qRT-PCR) was performed using a SYBR Green Realtime PCR Master Mix -Plus- (Toyobo). The quality was analysed on agarose gels, and the quantity was measured using a Varioskan Flash (Thermo Fisher Scientific, USA). Sequence-specific primers, listed in Additional file 8: Table S1, were synthesized by Invitrogen, and β-actin used as an endogenous reference gene. The real-time PCR was carried out by reverse transcription 1 μl of RNA, and the PCR amplification was performed as follows: 5 μl of 2 × SYBR green fluorescence quantitation reagent; 1 μl each of upstream and downstream primers, with a final concentration of 200 nmol/L; 1 μl of cDNA; 2 μl of reaction buffer. The amplification procedure was as follows: 95 °C for 5 min, 95 °C for 10 s, 60 °C for 35 s, repeated for 40 cycles. Samples were analysed in triplicate, and the melting curve was examined to verify that a single product was amplified. For quantitative analysis, all samples were analysed using the ΔΔCT value method.

### Enzyme-linked immunosorbent assay (ELISA)

Rat kidney tissues (100 mg) were homogenized and centrifuged, and the supernatant was collected. Avidin-biotin complex-ELISA was used according to the manufacturer’s protocol to determine the levels of TGF-β1. The ELISA kits were purchased from Xitang Biotechnology (Shanghai, China). All experiments were repeated at least three times.

### Western blot analysis

The total proteins from rat kidneys and NRK-49F and NRK-52E cells were collected, and the protein concentrations were determined using a bicinchoninic acid (BCA) protein assay kit (Beyotime). The proteins (20 μg) from each sample were separated by SDS-PAGE and transferred to a polyvinylidene difluoride membrane (Solarbio, Beijing, China). After treatment with 5% skim milk at 4 °C overnight, the membranes were incubated with various antibodies for 1 h and were then incubated with the appropriate horseradish peroxidase-conjugated secondary antibody (Beyotime). The bound antibodies were visualized by chemiluminescence detection on autoradiographic film. Quantification was performed by measurement of the signal intensity using Image-Pro Plus software (version 6.0, Media Cybernetics, Silver Spring, USA) and was normalized to that for the β-actin or GAPDH antibodies.

### NICD siRNA and overexpression

Short interfering RNAs against NICD or pcDNA3.1-NICD plasmid were transfected into NRK-52E or NRK-49F cells using Lipofectamine™ 2000 reagent (Invitrogen, Carlsbad, CA, USA) according to the manufacturer’s instructions. Sequence-specific primers synthesized by Invitrogen are listed in Additional file 9: Table S2.

### Statistical analysis

Data are presented as means ± standard deviations (SDs) of the means. All statistical analyses were performed using Statistical Package for the Social Sciences (version 16.0, SPSS Inc., Chicago, USA). A two-sided Student’s t-test was used to analyse differences between the two groups. One-way analysis of variance with Bonferroni’s posttest was used when more than two groups were present. A *p*-value of < 0.05 was considered statistically significant.

## Results

### Epithelial and interstitial Notch1 signalling is activated in kidneys following injury

First, we evaluated myofibroblastic phenotype and fibrosis in the obstructed kidneys. HE staining revealed marked tubular dilation and atrophy accompanied by widened interstitial space (Additional file 1: Figure S1A), and Masson trichrome staining indicated excessive deposition of total collagen (Additional file 1: Figure S1B). The fibrotic changes were also confirmed by excessive deposition of ECM components, including elevated protein expression of collagens I and III (Additional file 1: Figure S1C, F). Thus, as expected, ureteral obstruction induced interstitial fibrosis in kidneys. Furthermore, upregulated mRNA and protein expression levels of the mesenchymal markers α-SMA, vimentin and N-cadherin, and downregulated expression of the epithelial marker E-cadherin were observed, denoting the induction of EMT and myofibroblastic phenotype during fibrosis (Additional file 1: Figure S1D-F). In CKD patients, excessive collagen deposition and marked interstitial fibrosis were also observed (Additional file 1: Figure S1G), and were accompanied by EMT, as determined by enhanced expression of collagen I and reduced expression of E-cadherin (Additional file 1: Figure S1H). Previous studies have revealed a key role of TGF-β1 in fibrogenesis. Here, the levels of TGF-β1 and its receptor TGF-β1R were increased in the obstructive kidneys (Additional file 2: Figure S2A-C), which resulted in the activation of Smad2/3 downstream signalling (Additional file 2: Figure S2D). In addition, evidence from mRNA microarray analysis also supported the observation that enhanced expression of TGF-β family members was associated with matrix deposition in kidneys following injury, as confirmed by qRT-PCR (Additional file 2: Figure S2E, F).

**Fig. 1:**
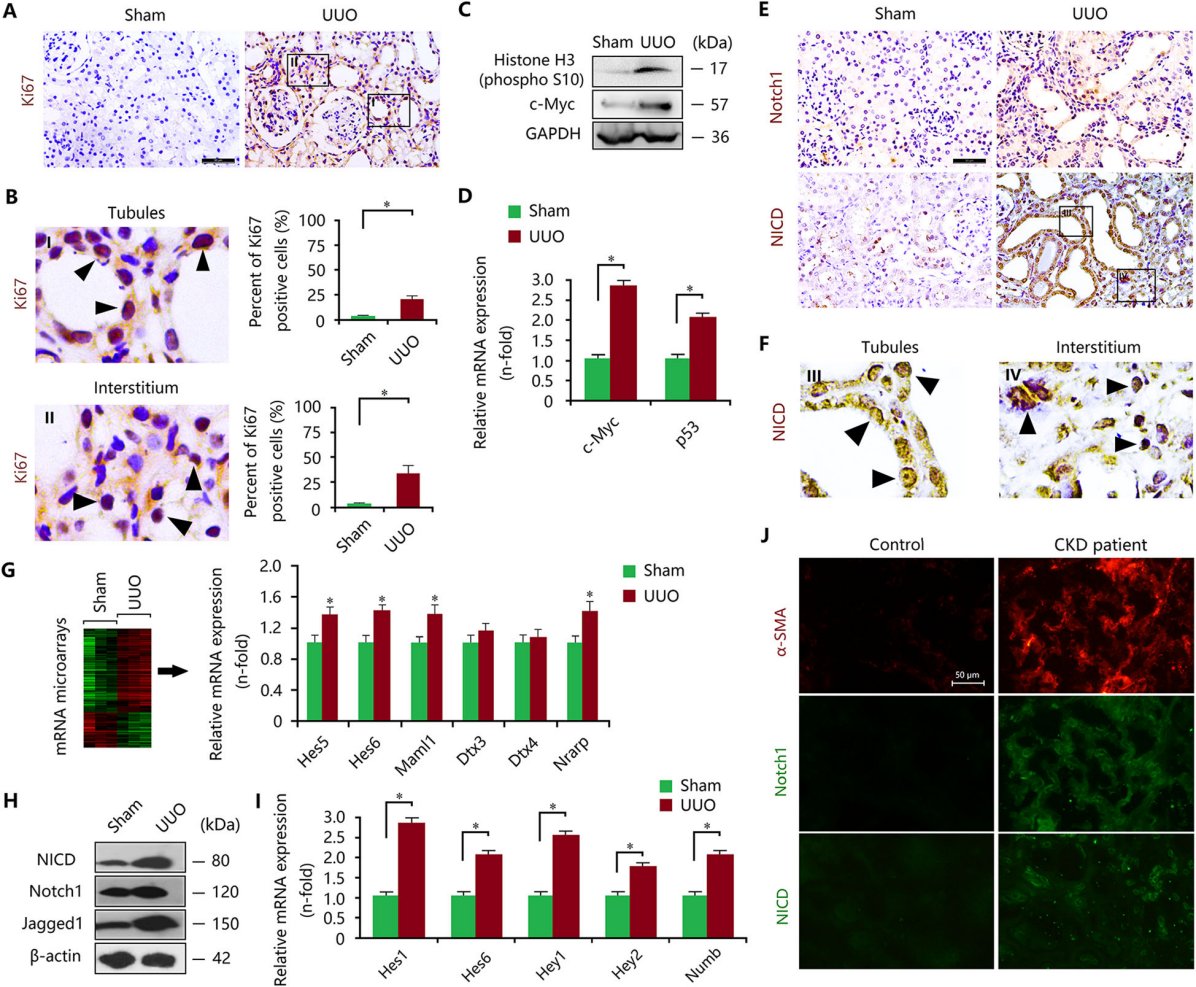
Epithelial and interstitial Notch1 activity is associated with the myofibroblastic phenotype in UUO kidneys. **a** Enhanced expression of Ki67 in UUO kidneys determined by immunohistochemical staining. Bar = 50 μm. **b** Upregulated Ki67 expression occurred in the tubules and interstitium of the kidney. **c** Increased expression of Histone H3 (phosphor S10) and c-Myc in UUO kidneys determined by western blot. **d** The mRNA expression levels of c-Myc and P53 were augmented in UUO kidneys determined by qRT-PCR. **e** Immunohistochemical staining showed that the expression levels of Notch1 and NICD were elevated in UUO kidneys. Bar = 50 μm. **f** NICD expression was upregulated in the tubules and interstitium of kidneys. **g** mRNA microarray revealed enhanced expression of Notch1-associated genes in UUO kidneys. **h** The expression levels of NICD, Notch1 and Jagged1 in UUO kidneys were increased. **i** Increased mRNA expression levels of Hes1, Hes6, Hey1, Hey2, and Numb in UUO kidneys were observed. **j** Elevated expression of Notch1, NICD and α-SMA determined by immunofluorescence staining in renal tubules and interstitium of CKD patients. All data are presented as means ± SDs. ^*^P < 0.05, ^**^P < 0.01 versus the sham group

Next, we investigated the activity of proliferation-associated Notch1 signalling in vivo. In the obstructed kidneys, enhanced expression of Ki67 was observed in the renal cortex (Fig. 1a), especially around renal tubules and in the interstitium (Fig. 1b), revealing a pathological manifestation of excessive proliferative TECs and fibroblasts during renal fibrosis. In addition, this kind of proliferative behaviour was also confirmed by elevated expression of phospho-Histone H3 (Ser10), c-Myc and p53 (Fig. 1c, d). This evidence suggested that myofibroblasts may originate not only from TECs by the EMT but also from interstitial fibroblasts. Multiple signalling pathways including Notch1 signalling may be responsible for this proliferation of TECs and interstitial fibroblasts. To clarify the activity and location of Notch1 signalling, immunohistochemical staining, western blot and qRT-PCR analyses were performed (Fig. 1e-i). We found that the expression levels of Notch1 and its intracellular domain NICD were enhanced and were mainly expressed in renal tubules and interstitium (Fig. 1e-f), consistent with Ki67-staining results. Aberrant activation of Notch1 signalling in the fibrotic kidneys was also confirmed by mRNA microarray (Fig. 1g) and was confirmed by western blot and qRT-PCR (Fig. 1h, i). In addition, we also evaluated the expression of Notch2, Notch3 and Notch4 in UUO kidneys. Although Notch2 and Notch4 expression is elevated in the UUO model compared to the Sham group, the difference is not so significant relative to Notch1 (Additional file 3: Figure S3). Based on these findings, Notch1 may be the most important factor to trigger fibrogenic responses in kidneys. In CKD patients, immunofluorescence staining revealed that Notch1 and α-SMA were co-expressed in the tubules and interstitium of the kidney during fibrosis (Fig. 1j), accompanied by enhanced expression of NICD, indicating that proliferation-associated Notch1 signalling was activated in the TECs and fibroblasts of kidney tissues.

To further clarify the role of Notch in the pathogenesis of fibrosis, DAPT, a γ-secretase inhibitor that blocks Notch1 signalling [26], was used in UUO kidneys. We found that DAPT inhibited UUO-induced kidney injury and excessive deposition of total collagen, as determined by HE and Masson staining (Additional file 4: Figure S4A, B), and decreased the expression of type III collagen, as determined by immunohistochemical staining (Additional file 4: Figure S4C). In addition, administration of DAPT also inhibited the mRNA expression of α-SMA, TGF-β1, and type I and III collagens (Additional file 4: Figure S4D).

Together, these results suggest that the activation of epithelial and interstitial Notch1 signalling may be involved in the formation of α-SMA-positive myofibroblasts and the development of fibrosis in kidneys following injury.

### Injury induces Notch1 signalling activation and myofibroblastic phenotype via the EMT and FMD in vitro

**Fig. 2:**
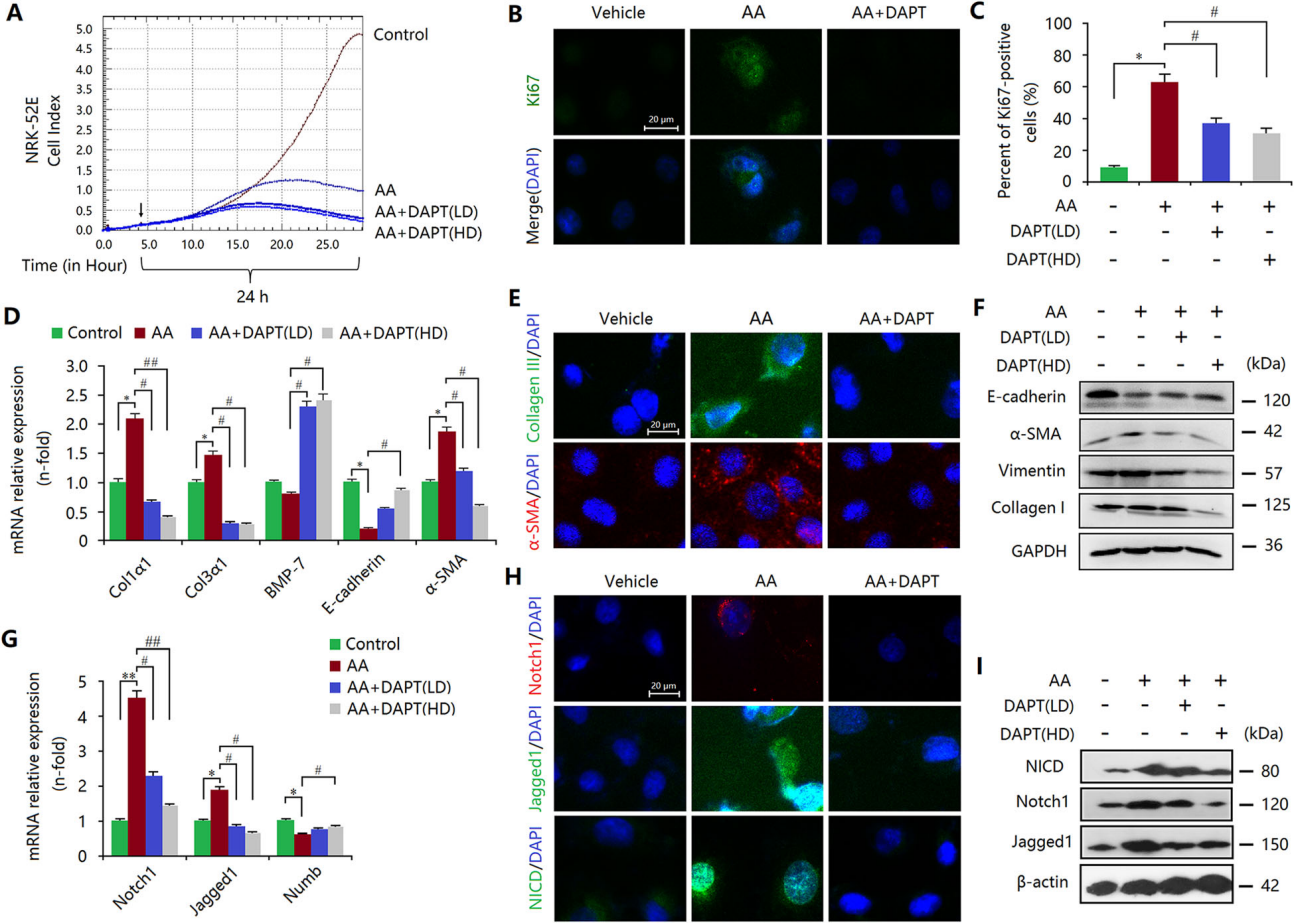
Notch1 signalling is activated in TECs after AA injury. **a** RTCA assay indicating the survival index of NRK-52E cells treated with AA with or without DAPT. **b** Upregulated Ki67 expression, determined by immunofluorescence staining, was inhibited by DAPT treatment. Bar = 20 μm. **c** The ratio of Ki67-positive cells in total cells according to the results of immunofluorescence staining. **d** qRT-PCR showed that increased mRNA expression levels of Col1α1, Col3α1 and α-SMA, and decreased mRNA levels of E-cadherin and BMP-7 in AA-treated NRK-52E cells were inhibited by DAPT treatment. **e** Elevated expression levels of α-SMA and collagen III in AA-treated NRK-52E cells were inhibited by DAPT treatment. Bar = 20 μm. **f** Increased expression levels of α-SMA, vimentin and collagen I, and decreased expression of E-cadherin in AA-treated NRK-52E cells were inhibited by DAPT treatment. **g** Increased mRNA expression levels of Notch1, Jagged1 and Numb in AA-treated NRK-52E cells were inhibited by DAPT treatment. **h** Elevated expression levels of Notch1, NICD and Jagged1 in AA-treated NRK-52E cells were inhibited by DAPT treatment. Bar = 20 μm. **i** Enhanced expression levels of Notch1, Jagged1 and NICD in AA-treated NRK-52E cells were inhibited by DAPT treatment. All data are presented as means ± SDs. AA, 10 ng/ml; DAPT (HD), high-dose (10 μmol/L); DAPT (LD), low-dose (1 μmol/L). ^*^P < 0.05, ^**^P < 0.01 versus the control; ^#^*P* < 0.01, ^#^*P* < 0.05 versus the AA-treated group

Our above in vivo evidence showed that epithelial and interstitial Notch1 signalling activation may contribute to the induction of myofibroblasts and fibrosis. However, these observations needed be confirmed again in vitro*.* Thus, we investigated the role of Notch1 signalling in cultured TECs (NRK-52E) and interstitial fibroblasts (NRK-49F) with the treatment of aristolochic acid (AA). Real-time cellular analysis (RTCA) assay revealed that AA significantly induced the injury of NRK-52E cells (Fig. 2a), which was associated with increases in apoptotic and necrotic cells [27, 28]. In addition, AA enhanced the percentage of Ki67-positive cells among total cells (Fig. 2b, c), which may be a feedback mechanism. Thus, these results indicated over-proliferation of TECs in AA-induced injury [26]. In this process, myofibroblast- and fibrosis-associated molecules were induced, and the marker levels of epithelial cells were reduced (Fig. 2d-f), suggesting that the injury-induced EMT programme may contribute to myofibroblastic phenotype and fibrosis. Further study revealed that this proliferative change of TECs was associated with Notch1 signalling activation, as demonstrated by increased protein expression (Notch1, Jagged1 and Numb) and enhanced nuclear transport of NICD (Fig. 2g-i). To elucidate the relationship between Notch1 signalling with the EMT programme, DAPT was used in cultured TECs (Fig. 2g-i). We found that DAPT also reduced AA-treated NRK-52E cell numbers (Fig. 2a). The decrease in cell number was associated with the inhibition of proliferation, as shown by the reduced percentage of Ki67-posive cells (Fig. 2b, c). As a result, DAPT inhibited epithelial phenotypes and acquired mesenchymal characteristic features (Fig. 2d-f), leading to excessive collagen accumulation. Therefore, epithelial Notch1 signalling-mediated EMT was crucial for myofibroblastic phenotype and fibrosis-like outcomes.

**Fig. 3:**
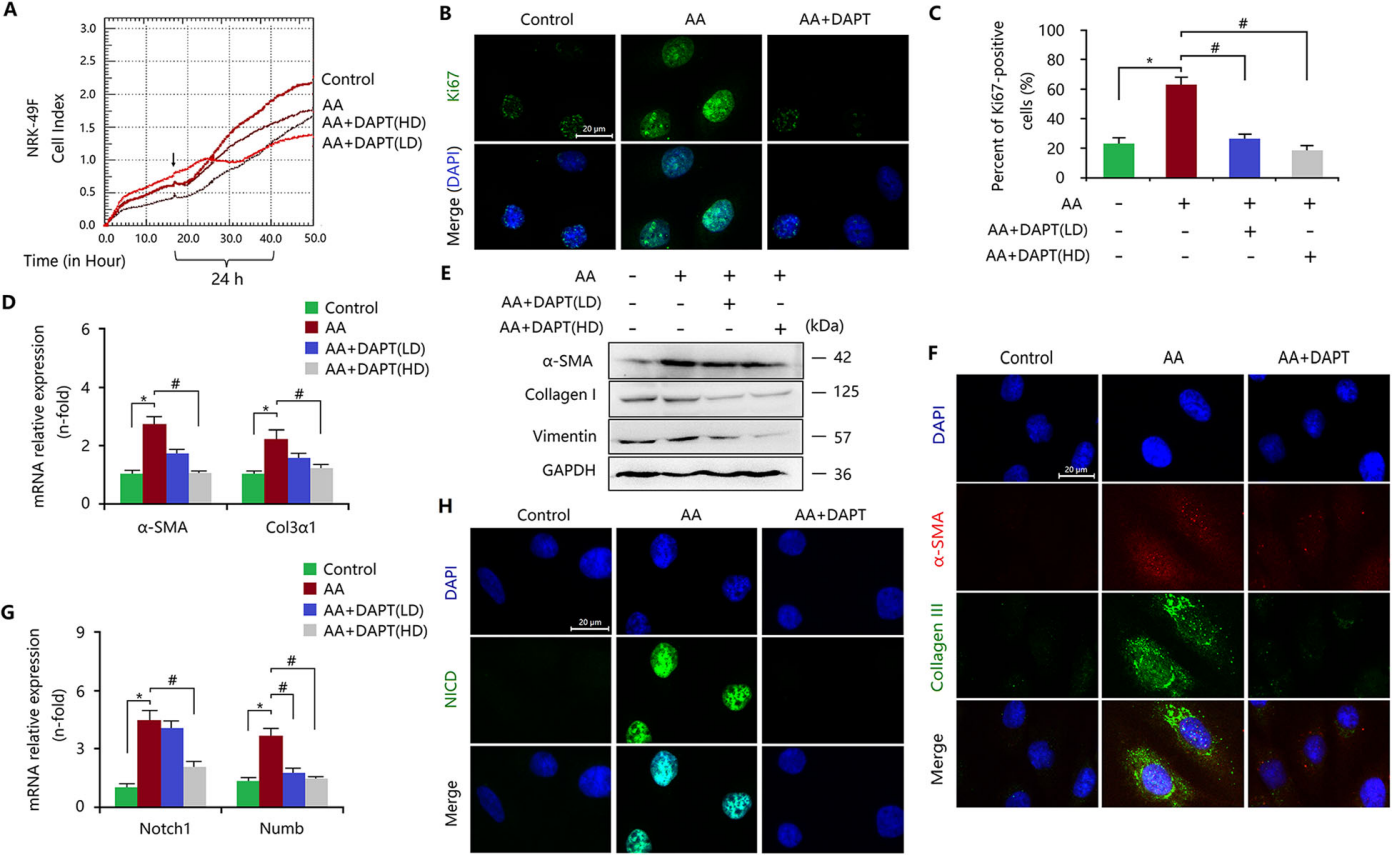
Notch1 signalling is activated in fibroblasts after AA injury. **a** RTCA assay indicated the survival index of NRK-49F cells treated with AA with or without DAPT. **b** Upregulated Ki67 expression, determined by immunofluorescence staining, was inhibited by DAPT treatment. Bar = 20 μm. **c** Ratio of Ki67-positive cells in total cells according to the results of immunofluorescence staining. **d** qRT-PCR showed that increased mRNA expression levels of Col3α1 and α-SMA in AA-treated NRK-49F cells were inhibited by DAPT treatment. **e** Increased expression levels of α-SMA, vimentin and collagen I in AA-treated NRK-49F cells were inhibited by DAPT treatment. **f** Elevated expression levels of α-SMA and collagen III in AA-treated NRK-49F cells were inhibited by DAPT treatment. Bar = 20 μm. **g** Increased mRNA expression levels of Notch1 and Numb in AA-treated NRK-49F cells were inhibited by DAPT treatment. **h** Elevated expression of NICD in AA-treated NRK-49F cells was inhibited by DAPT treatment. Bar = 20 μm. All data are presented as means ± SDs. AA, 10 ng/ml; DAPT (HD), high-dose (10 μmol/L); DAPT (LD), low-dose (1 μmol/L). ^*^P < 0.05 versus the control; ^#^*P* < 0.01 versus the AA-treated group

Similarly, in NRK-49F cells, AA induced the injury (Fig. 3a) and increased the percentage of Ki67-posive cells (Fig. 3b, c). However, the sensitivity of fibroblasts was weaker than that of epithelial cells when exposed to AA. In patients with AA-induced nephropathy (AAN), the injury of TECs may be more important than that of interstitial fibroblasts to trigger fibrosis, which are associated with direct drug contact and the susceptibility to mutation [26, 29]. In response to AA stimulus, the expression levels of α-SMA and ECM components in NRK-49F cells were also increased (Fig. 3d-f). Moreover, AA enhanced the activity of the Notch1 pathway by upregulating the expression levels of Notch1 and Numb and by inducing the nuclear localization of NICD (Fig. 3g, h). Thus, AA mediated FMD and myofibroblastic phenotype in fibroblasts through activating Notch1 signalling. Using the DAPT to inhibit the Notch1 pathway (Fig. 3g, h), the AA-mediated proliferation of NRK-49F cells was inhibited, though cell number did not change obviously (Fig. 3a, b). Furthermore, DAPT treatment inhibited and even reversed AA-induced FMD and myofibroblastic phenotype, resulting in the reduction of ECM accumulation (Fig. 3d, f).

Taken together, these results suggested a key role of proliferation-associated Notch1 signalling in fibrosis. During the process of injury, epithelial and interstitial Notch1 signalling was activated and then promoted myofibroblastic phenotype and ECM deposition via the EMT and FMD, respectively. Blockade of Notch1 signalling with DAPT can inhibit and even reverse to some extent the EMT and FMD programmes, reducing myofibroblastic phenotype and attenuating renal fibrosis.

### Notch1 mediates myofibroblastic phenotype by activating TGF-β1/Smad2/3 signalling

**Fig. 4:**
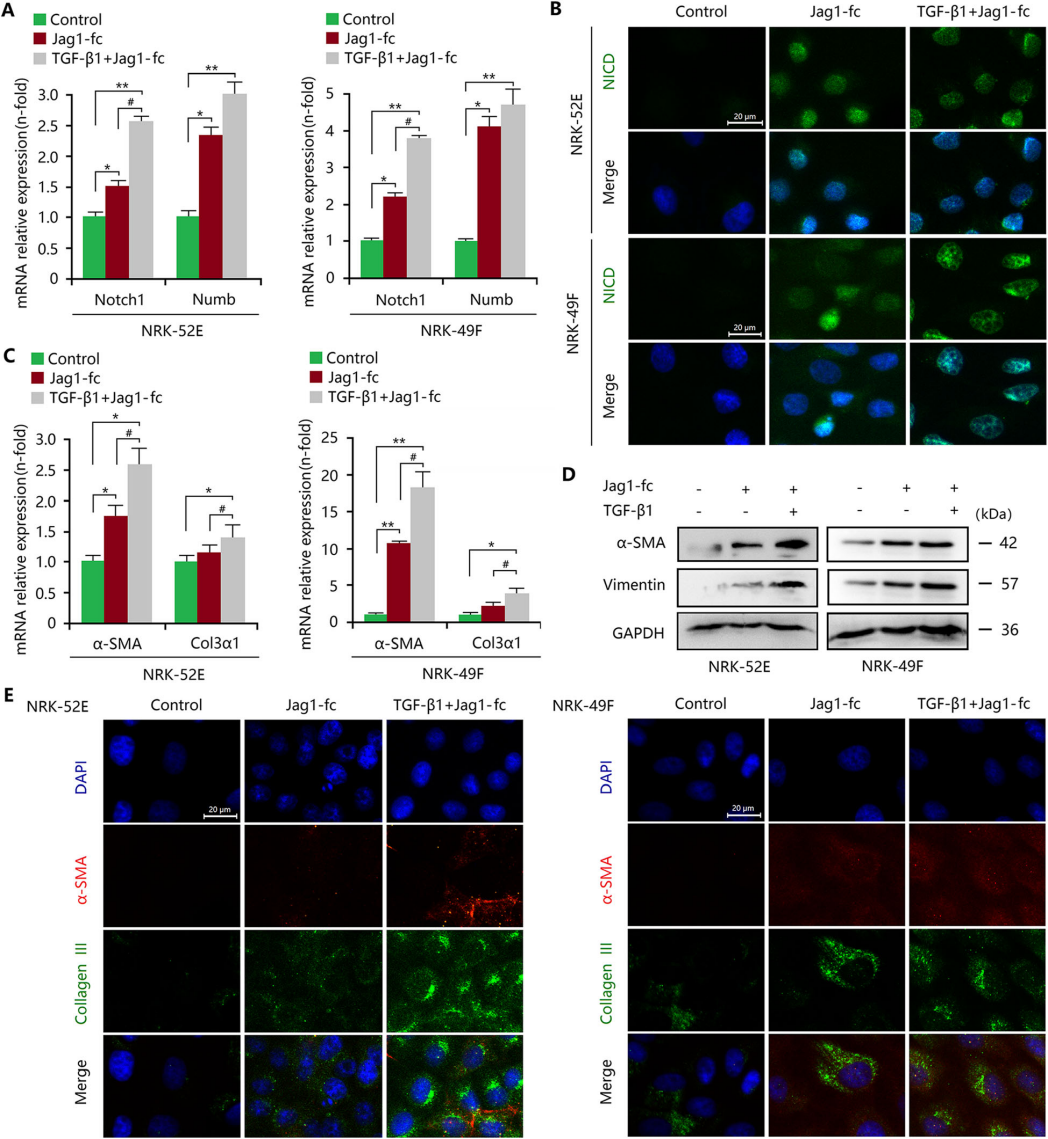
Activated Notch1 signalling induces myofibroblastic phenotype in vitro. **a** Increased mRNA expression levels of Notch1 and Numb in Jag1-fc-treated NRK-52E and NRK-49F cells were strengthened by TGF-β1. **b** Upregulated NICD expression and nuclear localization in Jag1-fc-treated NRK-52E and NRK-49F cells were strengthened by TGF-β1. Bar = 20 μm. **c** Increased mRNA expression levels of Col3α1 and α-SMA in Jag1-fc-treated NRK-52E and NRK-49F cells were enhanced by TGF-β1. **d** Increased expression of α-SMA and vimentin in Jag1-fc-treated NRK-52E cells or NRK-49F cells were enhanced by TGF-β1. **e** Elevated expression of α-SMA and collagen III in Jag1-fc-treated NRK-52E and NRK-49F cells was strengthened by TGF-β1. Bar = 20 μm. All data are presented as means ± SDs. Jag1-fc, 1 μg/ml; TGF-β1, 5 ng/ml. ^*^P < 0.05, ^**^P < 0.05 versus the control; ^#^*P* < 0.05 versus the Jag1-fc-treated group

To elucidate the role of epithelial and interstitial Notch1 activation in myofibroblastic phenotype regardless of the background of the damage, exogenous recombinant proteins TGF-β1 and Jag1-fc (a Notch1 ligand jagged 1) were used to investigate the role of activated Notch1 signalling in TECs and fibroblasts. As expected, qRT-PCR analysis showed that Jag1-fc significantly increased the mRNA expression of Notch1 and Numb in NRK-52E cells and NRK-49F cells (Fig. 4a). Immunofluorescence staining also confirmed Jag1-fc-induced activation of Notch1 signalling by upregulated expression and nuclear localization of NICD (Fig. 4b). As a result, Jag1-fc increased the expression of α-SMA, vimentin and collagen III in NRK-52E cells and NRK-49F cells (Fig. 4c-e). These results indicated that in vitro activation of Notch1 signalling contributed to myofibroblastic phenotype and ECM deposition in TECs and fibroblasts.

Interestingly, TGF-β1 enhanced significantly the activity of Notch1 signalling and it can be strengthened by Jag1-fc treatment in both NRK-52E cells and NRK-49F cells (Fig. 4a, b), indicating that there are a dialogue mechanism between Notch1 and TGF-β1 signalling. Previous studies have shown that TGF-β1 treatment alone can induce myofibroblastic phenotype and increase ECM levels [30]. Here, our results revealed that compared with TGF-β1 treatment alone, the combination of TGF-β1 and Jag1-fc markedly promoted the expression of α-SMA, vimentin and collagen III in NRK-52E cells and NRK-49F cells (Fig. 4c-e), revealing that Notch1 signalling and TGF-β1 signalling may be dependent and drive together the formation of myofibroblastic phenotype and fibrosis.

**Fig. 5:**
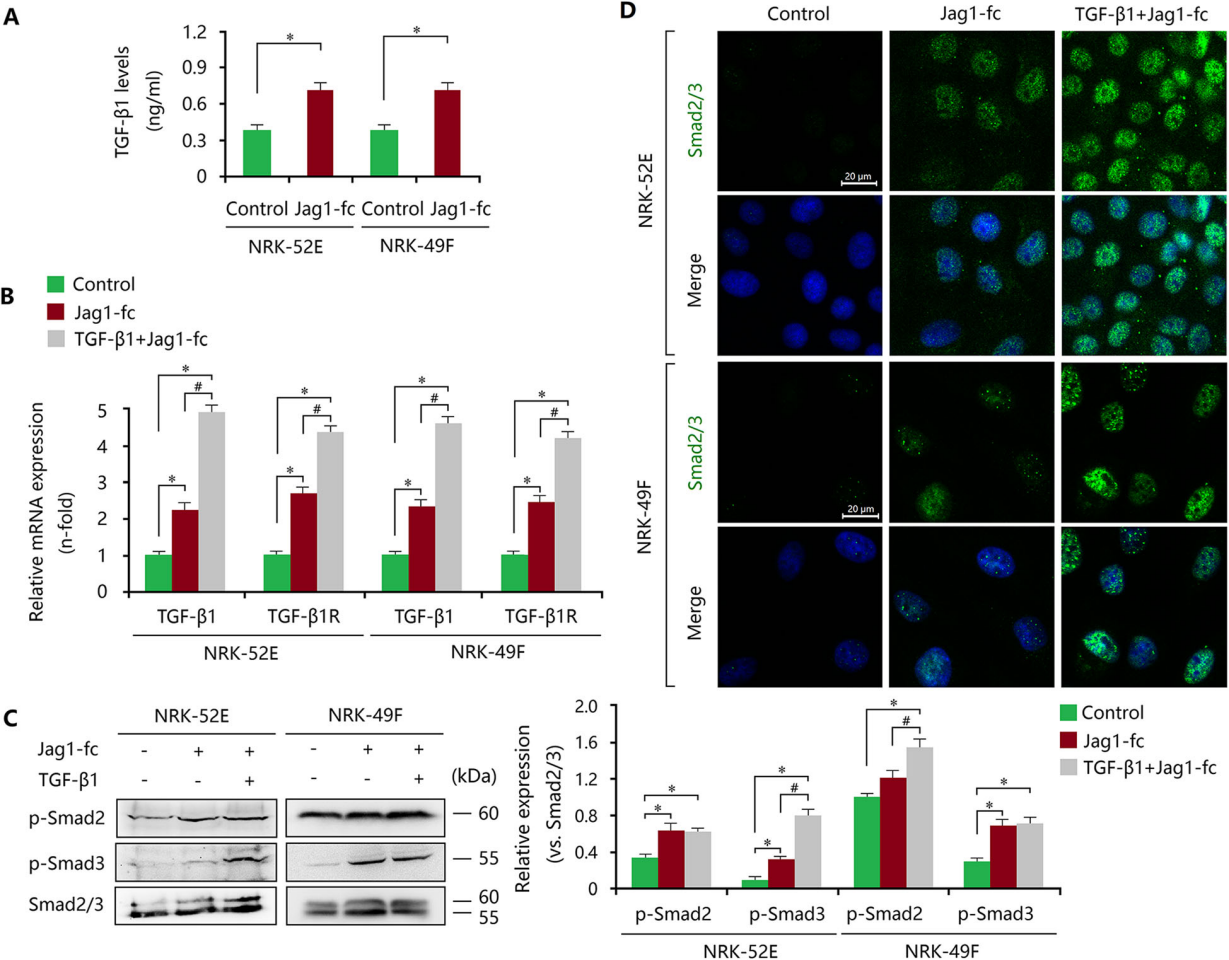
Notch1 signalling induces EMT and FMD by activating the TGF-β1/Smad2/3 pathway in vitro. **a** ELISA assay revealed that the levels of TGF-β1 in NRK-52E and NRK-49F cells were increased by Jag1-fc treatment. **b** Upregulated mRNA expression levels of TGF-β1 and TGF-β1R in Jag1-fc-treated NRK-52E and NRK-49F cells were strengthened by TGF-β1. **c** Increased expression and phosphorylation of Smad2 and Smad3 in Jag1-fc-treated NRK-52E and NRK-49F cells were enhanced by TGF-β1. **d** Upregulated Smad2/3 expression and nuclear localization in Jag1-fc-treated NRK-52E and NRK-49F cells were strengthened by TGF-β1. Bar = 20 μm. All data are presented as means ± SDs. Jag1-fc, 1 μg/ml; TGF-β1, 5 ng/ml. ^*^P < 0.05, versus the control; ^#^*P* < 0.05 versus the Jag1-fc-treated group

To further clarify the role of TGF-β1 during Notch1 signalling activation, TGF-β1 receptor (TGF-β1R) and downstream Smad2/3 signalling were analysed. We found that Jag1-fc treatment alone increased TGF-β1 levels in both NRK-52E and NRK-49F cells (Fig. 5a). In addition, Jag1-fc induced TGF-β1R expression, which could be strengthened by TGF-β1 (Fig. 5b). Further study showed that Jag1-fc induced the phosphorylation of p-Smad2 and p-Smad3 and the nuclear localization of Smad2/3 (Fig. 5c, d). This induction of Smad2/3 signalling was strengthened by TGF-β1 (Fig. 5c, d). In AA-treated NRK-52E or NRK-49F cells, DAPT treatment reduced the mRNA expression of TGF-β1 and TGF-β1R (Additional file 5: Figure S5A, B), thereby inhibiting the phosphorylation of p-Smad2 and p-Smad3 and the nuclear localization of Smad2/3 (Additional file 6: Figure S6A-D). Therefore, these findings indicate that epithelial and interstitial Notch1 activation contribute to myofibroblastic phenotype by inducing the TGF-β1/Smad2/3 signalling cascade, and that TGF-β1 may also have a dialogue with Notch1 signalling to trigger fibrosis.

### TGF-β1 induces myofibroblastic phenotype dependent on Notch1 signalling

**Fig. 6:**
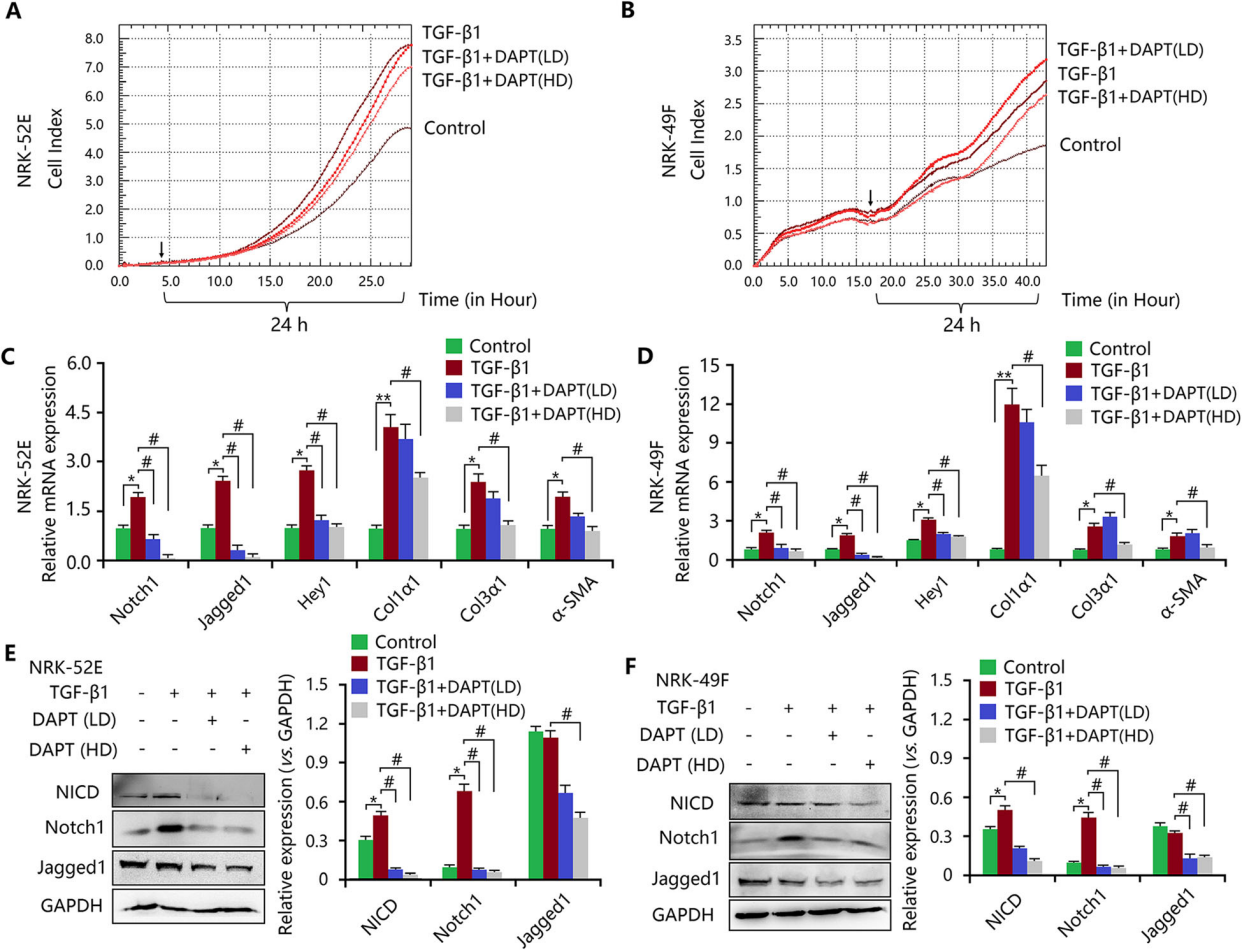
TGF-β1 induces Notch1 signalling activation during the EMT and FMD in vitro. **a** RTCA assay indicated the survival index of NRK-52E cells treated with TGF-β1 with or without DAPT. **b** RTCA assay indicated the survival index of NRK-49F cells treated with TGF-β1 with or without DAPT. **c** Increased mRNA expression levels of Notch1, Jagged1, Hey1, Col1α1, Col3α1, and α-SMA in TGF-β1-treated NRK-52E cells were inhibited by DAPT. **d** Increased mRNA expression levels of Notch1, Jagged1, Hey1, Col1α1, Col3α1, and α-SMA in TGF-β1-treated NRK-49F cells were inhibited by DAPT. **e** Elevated expression levels of NICD, Notch1 and Jagged1 in TGF-β1-treated NRK-52E cells were inhibited by DAPT. **f** Elevated expression levels of NICD, Notch1 and Jagged1 in TGF-β1-treated NRK-49F cells were inhibited by DAPT. All data are presented as means ± SDs. TGF-β1, 5 ng/ml. DAPT (HD), high-dose (10 μmol/L); DAPT (LD), low-dose (1 μmol/L). ^*^P < 0.05, versus the control; ^#^*P* < 0.05 versus the TGF-β1-treated group

**Fig. 7:**
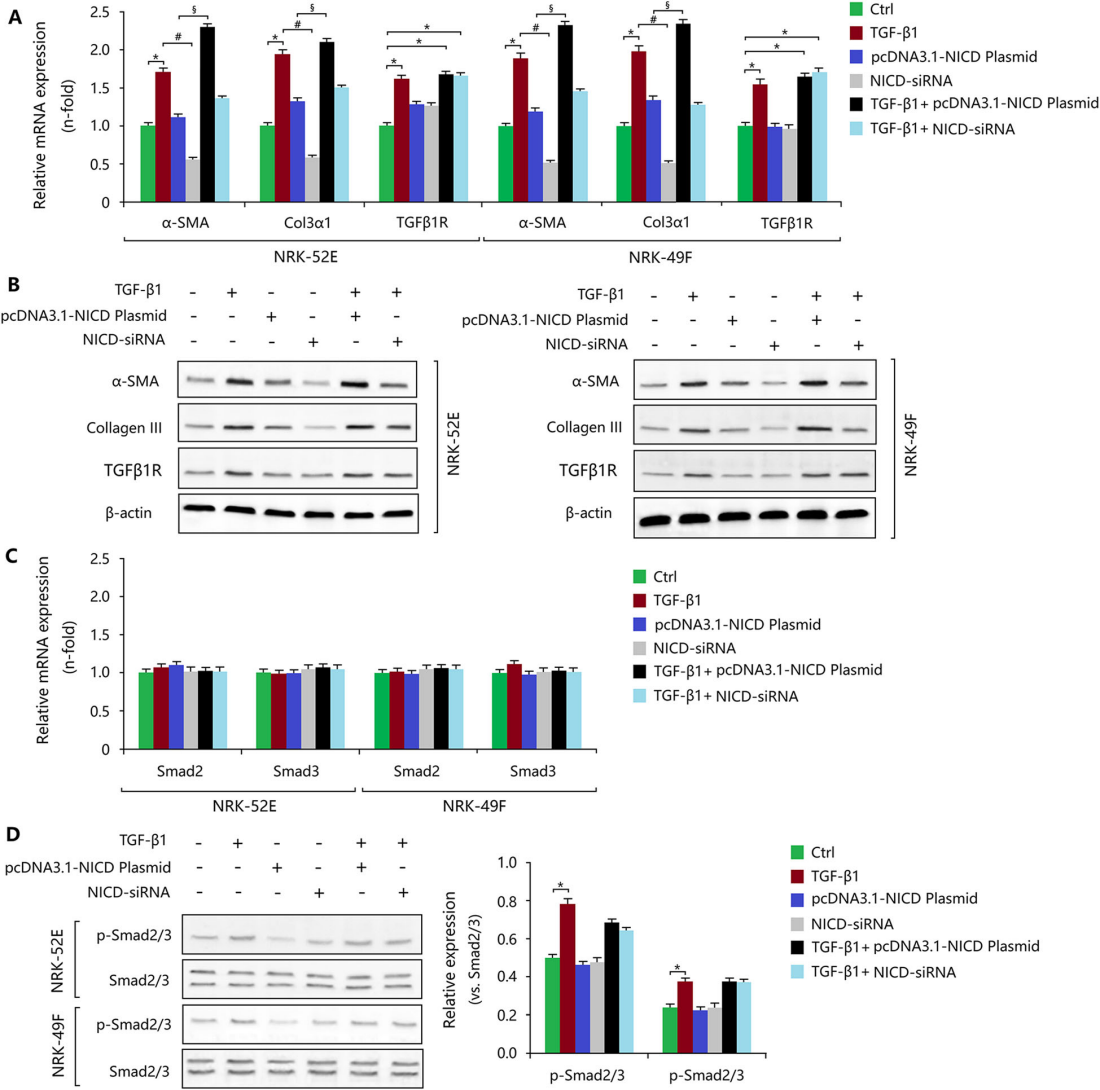
Notch1 activity mediates myofibroblastic phenotype dependent on TGF-β1 in vitro. **a** The mRNA expression levels of TGF-β1R, Col3α1 and α-SMA in NRK52E and NRK-49F cells treated with TGF-β1 with or without pcDNA3.1-NICD plasmid or NICD siRNA. **b** The protein expression levels of TGF-β1R, α-SMA and collagen III in NRK52E and NRK-49F cells treated with TGF-β1 with or without pcDNA3.1-NICD plasmid or NICD siRNA. **c** The mRNA expression levels of Smad2 and Smad3 in NRK52E and NRK-49F cells treated with TGF-β1 with or without pcDNA3.1-NICD plasmid or NICD siRNA. **d** The protein expression and phosphorylation levels of Smad2 and Smad3 in NRK52E and NRK-49F cells treated with TGF-β1 with or without pcDNA3.1-NICD plasmid or NICD siRNA. All data are presented as means ± SDs. TGF-β1, 5 ng/ml. ^*^P < 0.05, versus the control; ^#^*P* < 0.05 versus the TGF-β1-treated group; ^§^*P* < 0.05 versus the pcDNA3.1-NICD plasmid-treated group

As stated before, injury-induced Notch1 signalling activation resulted in the induction of myofibroblastic phenotype via TGF-β1/Smad2/3 signalling. Here, we investigated the activity of Notch1 signalling in response to TGF-β1 stimulus. We found that TGF-β1 induced cellular proliferation of NRK-52E and NRK-49F cells (Fig. 6a, b). This proliferative behaviour was inhibited by the small molecule DAPT (Fig. 6a, b). In addition, TGF-β1 promoted the expression of ECM components and induced myofibroblastic phenotype, and DAPT treatment significantly reduced TGF-β1-mediated fibrotic changes (Fig. 6c, d). Moreover, TGF-β1 enhanced the mRNA expression of Notch1, Jagged1 and Hey1 (Fig. 6c, d), and the protein expression of Notch1, Jagged and NICD, though these effects were inhibited by DAPT (Fig. 6e, f). Thus, TGF-β1 stimulated the activation of Notch1 signalling. To further confirm this hypothesis, NICD over-expression with pcDNA 3.1-NICD plasmid and siRNA knockdown were induced in NRK-52E or NRK-49F cells. As shown in Additional file 7: Figure S7A-C, pcDNA 3.1-NICD plasmid significantly increased NICD mRNA and protein expression levels and siRNA decreased NICD expression in both NRK-52E and NRK-49F cells. Treatment with TGF-β1 enhanced the expression of NICD when compared with the control. Moreover, combination of TGF-β1 and pcDNA 3.1-NICD plasmid also increased NICD expression. These results indicated that in injury-induced Notch1 activation, TGF-β1 was also activated to exert a stronger profibrotic effect. As a result, TGF-β1, as well as activation of Notch1, led to myofibroblastic phenotype and fibrotic outcomes (Fig. 7a, b). These changes were associated with the phosphorylation of Smad2/3 (Fig. 7c, d).

In short, these findings indicated that injury-induced epithelial and interstitial Notch1 activation contributes to the myofibroblastic phenotype and fibrosis through the EMT in TECs and the FMD in fibroblasts by targeting downstream TGF-β1/Smad2/3 signalling. Similarly, TGF-β1-mediated myofibroblastic phenotype and fibrotic outcomes were dependent on Notch1 activation, revealing a crosstalk role of Notch1 signalling with TGF-β1 in fibrogenesis following injury.

## Discussion

This study provides evidence that in vivo Notch1 signalling was associated with over-proliferation of TECs and interstitial fibroblasts during fibrosis in kidney tissues following injury. In vitro, Notch1 signalling was also activated to induce the myofibroblastic phenotype via the EMT in TECs and the FMD in fibroblasts after AA injury, but these effects were abolished by the Notch1 signalling inhibitor cyclopamine. Moreover, exogenous Jag-fc recombinant protein was used to activate Notch1 signalling, resulting in myofibroblastic phenotype and fibrotic outcomes. Thus, the epithelial and interstitial Notch1 signalling pathway played an important role in the formation of myofibroblastic phenotype by inducing the EMT and FMD during kidney injury.

As a key development-associated pathway, Notch1 signalling has been reported to be involved in cell fate determination, cell lineage specification and cell lineage stabilization [31]. Due to the crucial roles of Notch1 signalling in the formation of nephrons and in kidney development [32–34], some studies have hypothesized that aberrant activation of this signalling most likely leads to interstitial fibrosis [14, 19, 35]. Using pharmacologic, genetic, in vivo, and in vitro experiments, Bielesz and colleagues showed that activation of the Notch1 signalling pathway in TECs in patients and in mouse models of interstitial fibrosis is necessary for fibrosis development [19]. In addition, in kidneys with ureteral obstruction, Notch1 signalling was activated and then induced fibrosis, but this induction was inhibited by the Notch1 inhibitor dibenzazepine (DBZ) [14, 36]. These findings indicate that Notch1 has an initiating function for fibrosis development, which are also supported by our results from in vivo and in vitro models and in CKD kidneys. Moreover, we found that the signalling receptor Notch1 and its intracellular domain NICD were mainly expressed in renal tubules and interstitium, suggesting that epithelial and interstitial Notch1 signalling contributes to fibrosis. Further study also revealed that injury-induced Notch1 signalling activation occurred not only in cultured TECs but also in fibroblasts. Inhibition of Notch1 signalling with DAPT resulted in the reduction of myofibroblastic phenotype and fibrosis. In TECs, Notch1 induces myofibroblastic phenotype and ECM deposition by mediating the EMT [14, 19]. Similarly, Notch1 drives myofibroblastic phenotype in fibroblasts by inducing the FMD [37, 38]. This evidence supports a key role of epithelial and interstitial Notch1 signalling in the induction of myofibroblastic phenotype in kidneys following injury.

During fibrosis, EMT and FMD progress can be induced by TGF-β1 [30]. The TGF-β signal activates Smad2 and Smad3 via a tetrameric complex type I and type II receptor and then binds to Smad4. The Smad complex transfers to the nucleus together with transcription factors (e.g., TBP-2) to inhibit the expression of the marker proteins of epithelial cells (e.g., E-cadherin) and to induce the expression of proteins of mesenchymal cells (e.g., α-SMA), thereby promoting EMT [39, 40]. Similarly, TGF-β1 is a potent driver of myofibroblast differentiation from resident fibroblasts [3, 41]. In response to TGF-β1 stimuli, fibroblasts become activated and acquire a migratory phenotype, termed proto-myofibroblasts. Proto-myofibroblasts are characterized by the presence of stress fibres containing filamentous actin, and the synthesis of ED-A fibronectin. In the presence of TGF-β1, further differentiation occurs to a contractile phenotype, termed a differentiated myofibroblast, characterized by the expression of α-SMA [42]. In the present study, we identified enhanced levels of TGF-β1 in the obstructive kidneys, as well as AA-treated TECs and fibroblasts. Further study indicated that TGF-β1 mediates the EMT and FMD by inducing the phosphorylation and nuclear localization of Smad2/3. Thus, TGF-β1 and downstream cascade Smad2/3 signalling contribute to the EMT and FMD progress and fibrosis in kidneys following injury.

Considering that both Notch1 and TGF-β1 are involved in the induction of myofibroblastic phenotype, it is important to determine whether there is a connection between TGF-β1 and Notch1 signalling in fibrosis. In kidneys after obstructive injury, Notch1 signalling triggers fibrosis by activating the TGF-β1 pathway [14]. Increased epithelial expression of Notch1 was associated with increased levels of TGF-β1, which thereby stimulates myofibroblast activation following epithelial injury [19]. In addition, Notch1 signalling was also reported inducing EMT via TGF-β1 production [43]. These results indicate that Notch1 signalling drives fibrosis by activating the TGF-β1/Smad2/3 pathway cascade and TGF-β1 may be a downstream molecule of Notch1 signalling. Conversely, our results showed that TGF-β1 also induced the activation of Notch1 signalling in TECs and fibroblasts. Morrissey and colleagues showed that TGF-β1 induces renal epithelial jagged-1 expression in fibrotic disease [44]. Functional integration of the TGF-β1/Smad and Notch1 pathways mediates the EMT [43] and also induces endothelial-mesenchymal transition (EndoMT) dependent of matrix metalloproteinase-9 (MMP-9) [45]. These findings reveal a crosstalk between TGF-β1 and Notch1 signalling in the induction of myofibroblastic phenotype and the development of fibrosis.

Given the importance of Notch1 signalling in the induction of myofibroblastic phenotype via the EMT and FMD, blockade of the Notch1 signalling may have an anti-fibrotic effect. DBZ is a potent and specific inhibitor of γ-secretase that leads to the progressive accumulation of APPL CTF fragments and a decrease in NICD production in a strictly dose-dependent manner [46]. Studies revealed that in vivo, DBZ markedly prevents Ang II-stimulated accumulation of macrophages and CD4^+^ T cells, which ERK-mediated angiogenesis simultaneously reverses the Th2 response [47]. In kidneys, DBZ treatment in vivo and in vitro blocks activated Notch1 signalling and markedly attenuates fibrosis and the expression of fibrotic markers, including collagens 1α1/3α1, fibronectin, and α-SMA [14, 19]. DAPT, another γ-secretase inhibitor, is a multimeric membrane protein complex that indirectly inhibits the activity of the γ-secretase substrate Notch1, which in turn affects cell signalling and cell differentiation [48]. Notch1 signalling is implicated in hepatic fibrogenesis and DAPT treatment has a protective effect on hepatocytes and ameliorates liver fibrosis [49]. Inhibition of the Notch1 signalling pathway with DAPT also prevents cholestatic liver fibrosis by decreasing the differentiation of hepatic progenitor cells into cholangiocytes [50]. In this study, we revealed that DAPT treatment not only markedly reduced the injury-induced myofibroblastic phenotype and fibrosis via inhibiting the EMT and FMD but also exerted its inhibitory effect on TGF-β1-mediated fibrotic changes, identifying a crosstalk between Notch1 and TGF-β1 in renal fibrosis. Thus, blockade of Notch1 signalling with DAPT or DBZ can be used as potential therapies for fibrotic kidney diseases [23, 35].

However, there are obvious limitations in this study, as the Notch1 signalling pathway in the kidneys was not induced to be over-activated (transgenic models) or knocked down (conditional gene knockout models) in vivo; therefore, it cannot be proved that Notch1 signalling is necessary to induce renal fibrosis. In addition, the dialogue between Notch1 and TGF-β1 and its molecular mechanism (e.g., direct or indirect mode of action) in this study need to be clarified. Thus, it is very important to investigate the crosstalk of Notch1 and TGF-β1 in the induction of myofibroblastic phenotype and the development of fibrosis.

## Conclusions

**Fig. 8:**
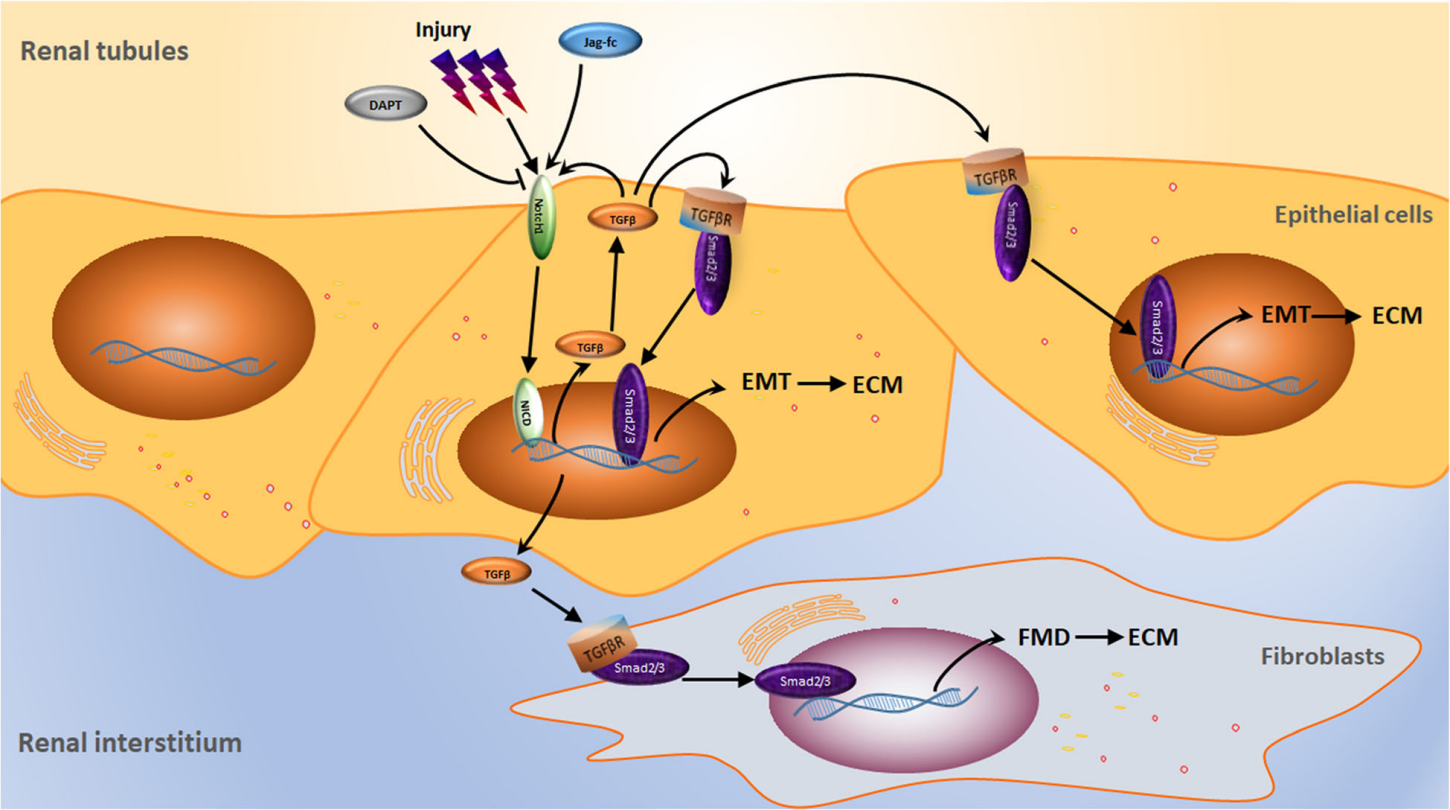
Injury-induced epithelial and interstitial Notch1 activity contributes to myofibroblastic phenotype and fibrosis. Injury stimulates the activation of the Notch1 signalling pathway in TECs and fibroblasts. Activated Notch1 signalling starts with a TGF-β1/Smad2/3 signalling cascade, which induces the EMT and FMD response, promotes myofibroblastic phenotype and ECM deposition, and results in interstitial fibrosis. Pharmacologic or genetic blockade of Notch1 signalling decreases TGF-β1 expression and abolishes injury-mediated EMT/FMD, myofibroblastic phenotype and fibrosis

Injury stimulates the activation of the Notch1 signalling pathway in TECs and fibroblasts. Activated Notch1 signalling starts with a TGF-β1/Smad2/3 signalling cascade, which induces the EMT and FMD response, promotes myofibroblastic phenotype and ECM deposition, and results in interstitial fibrosis (Fig. 8). Pharmacologic or genetic blockade of Notch1 signalling abolishes injury-mediated EMT/FMD, myofibroblastic phenotype, and decreased TGF-β1 expression and ECM production. Therefore, epithelial and interstitial Notch1 signalling may contribute to renal fibrosis via the induction of EMT/FMD, and inhibition of Notch1 signalling may have a therapeutic role for fibrotic kidney diseases.

## Supplementary information

**Additional file 1: Figure S1.** Myofibroblastic phenotype and fibrosis in UUO kidneys. (A) HE staining showed obvious kidney injury in UUO kidneys, and Masson’s trichrome staining revealed excessive deposition of total collagen. Bar = 100 μm. (B) Ureteral obstruction induced marked increases in interstitial damage and interstitial collagen deposition in kidneys of rats. (C) Increased expression of collagen III in UUO kidneys compared with the sham group. Bar = 100 μm. (D) Increased mRNA expression levels of α-SMA and vimentin in UUO kidneys compared with the sham group. (E) Increased expression of vimentin in UUO kidneys compared with the sham group. Bar = 100 μm. (F) Increased protein expression of vimentin, N-cadherin, α-SMA, and collagen I, and decreased expression of E-cadherin in UUO kidneys compared with the sham group. (G) HE and Masson’s trichrome staining showed obvious tubular and interstitial damage and excessive collagen deposition in CKD patients. Bar = 100 μm. (H) Increased expression of collagen I and decreased expression of E-cadherin in CKD patients. Bar = 50 μm. All data are presented as means ± SDs. ^*^P < 0.05, ^**^P < 0.01 versus the sham group.

**Additional file 2: Figure S2.** TGF-β1/Smad2/3 signalling is activated during fibrosis in UUO kidneys. (A) The expression of TGF-β1 in UUO kidneys was significantly increased. Bar = 100 μm. (B) ELISA assay revealed enhanced levels of TGF-β1 in UUO rats. (C) The expression levels of TGF-β1 and TGF-β1R in UUO kidneys were significantly increased. (D) The expression and location of Smad2/3 in UUO rats. Bar = 50 μm. (E) mRNA microarrays revealed that the expression levels of TGF-β1- and ECM-associated genes were enhanced in UUO rats. (F) The mRNA expression levels of TGF-β1, TGF-β1R, Col1α1, and Col3α1 were increased in UUO kidneys compared with those in the sham. All data are presented as means ± SDs. ^*^P < 0.05, ^**^P < 0.01 versus the sham group.

**Additional file 3: Figure S3.** The expression of Notch2, Notch3, and Notch4 in renal fibrosis. Increased protein expression of Notch2 and Notch4 in UUO kidneys compared with the sham group. However, the expression of Notch3 did not show any difference. All data are presented as means ± SDs. ^*^P < 0.05 versus the sham group.

**Additional file 4: Figure S4.** Inhibition of Notch1 signalling with DAPT reduces fibrosis. (A) Kidney injury and excessive deposition of total collagen in UUO kidneys were reduced by DAPT administration. Bar = 100 μm. (B) The interstitial damage and interstitial collagen deposition in UUO kidneys were evaluated according to HE and Masson’s trichrome staining. (C) Increased expression of type III collagen in UUO kidneys was inhibited by DAPT treatment. Bar = 100 μm. (D) qRT-PCR showed that increased mRNA expression levels of α-SMA, TGF-β1, Col1α1, and Col3α1 in UUO kidneys were inhibited by DAPT treatment. All data are presented as means ± SDs. ^*^P < 0.05 versus the control group; ^#^*P* < 0.05 versus the AA-treated group.

**Additional file 5: Figure S5.** The TGF-β1 signalling activity in TECs and fibroblasts following AA injury. (A) qRT-PCR showed that increased mRNA expression levels of TGF-β1 and TGF-β1R in AA-treated NRK-52E cells were inhibited by DAPT treatment. (B) qRT-PCR showed that increased mRNA expression levels of TGF-β1 and TGF-β1R in AA-treated NRK-49F cells were inhibited by DAPT treatment. AA, 10 ng/ml; DAPT (HD), high-dose (10 μmol/L); DAPT (LD), low-dose (1 μmol/L). All data are presented as means ± SDs. ^*^P < 0.05 versus the control group; ^#^*P* < 0.05 versus the AA-treated group.

**Additional file 6: Figure S6.** The Smad2/3 signalling activity in TECs and fibroblasts following AA injury. (A) Immunofluorescence staining indicated upregulated expression and nuclear localization of Smad2/3 in NRK-52E cells after AA treatment, but these effects were inhibited by DAPT. Bar = 20 μm. (B) Immunofluorescence staining indicated upregulated expression and nuclear location of Smad2/3 in NRK-49F cells after AA treatment, but it was inhibited by DAPT. Bar = 20 μm. (C) Upregulated phosphorylation levels of Smad2 and Smad3 in AA-treated NRK-52E cells were inhibited by DAPT. (D) Upregulated phosphorylation levels of Smad2 and Smad3 in AA-treated NRK-49F cells were inhibited by DAPT. AA, 10 ng/ml; DAPT (HD), high-dose (10 μmol/L); DAPT (LD), low-dose (1 μmol/L). All data are presented as means ± SDs. ^*^P < 0.05 versus the control group; ^#^*P* < 0.05 versus the AA-treated group.

**Additional file 7: Figure S7.** The NICD activity in TECs and fibroblasts treated with TGF-β1, pcDNA3.1-NICD plasmid, or NICD siRNA. (A) The expression of NICD in NRK-52E cells treated with TGF-β1, pcDNA3.1-NICD plasmid, or NICD siRNA. (B) The expression of NICD in NRK-49F cells treated with TGF-β1, pcDNA3.1-NICD plasmid, or NICD siRNA. (C) The mRNA expression of NICD in NRK-52E and NRK-49F cells treated with TGF-β1, pcDNA3.1-NICD plasmid, or NICD siRNA. All data are presented as means ± SDs. TGF-β1, 5 ng/ml. ^*^P < 0.05, versus the control group; ^#^*P* < 0.05 versus the TGF-β1-treated group; ^§^*P* < 0.05 versus the pcDNA3.1-NICD plasmid-treated group.

**Additional file 8: Table S1.** Two-step real-time RT-PCR primers for analysis.

**Additional file 9: Table S2.** Primers for NICD siRNA and overexpression.

## Data Availability

The datasets used and/or analysed during the current study are available from the corresponding author on reasonable request.

## Abbreviations

AA: Aristolochic acid
BMP-7: Bone morphogenetic protein-7
CKD: Chronic kidney disease
ECM: Extracellular matrix
ELISA: Enzyme linked immunosorbent assay
EMT: Epithelial-mesenchymal transition
FBS: Fetal bovine serum
FMD: Fibroblast-myofibroblast differentiation
HE: Hematoxylin and Eosin
Jag1-fc: Jagged-1-Fc
qRT-PCR: quantitative real-time polymerase chain reaction
RTCA: Real-time cellular analysis
SD: Standard deviation
TECs: Tubular epithelial cells
TGF-β1: Transforming growth factor-β1
UUO: Unilateral ureteral obstruction
α-SMA: alpha-smooth muscle actin
