# Supplementary figures and images for: Epithelial and interstitial Notch1 activity contributes to the myofibroblastic phenotype and fibrosis
Source: Cell Commun Signal. 2019 Nov 12;17:145. doi: 10.1186/s12964-019-0455-y (PMC6849313; doi:10.1186/s12964-019-0455-y)

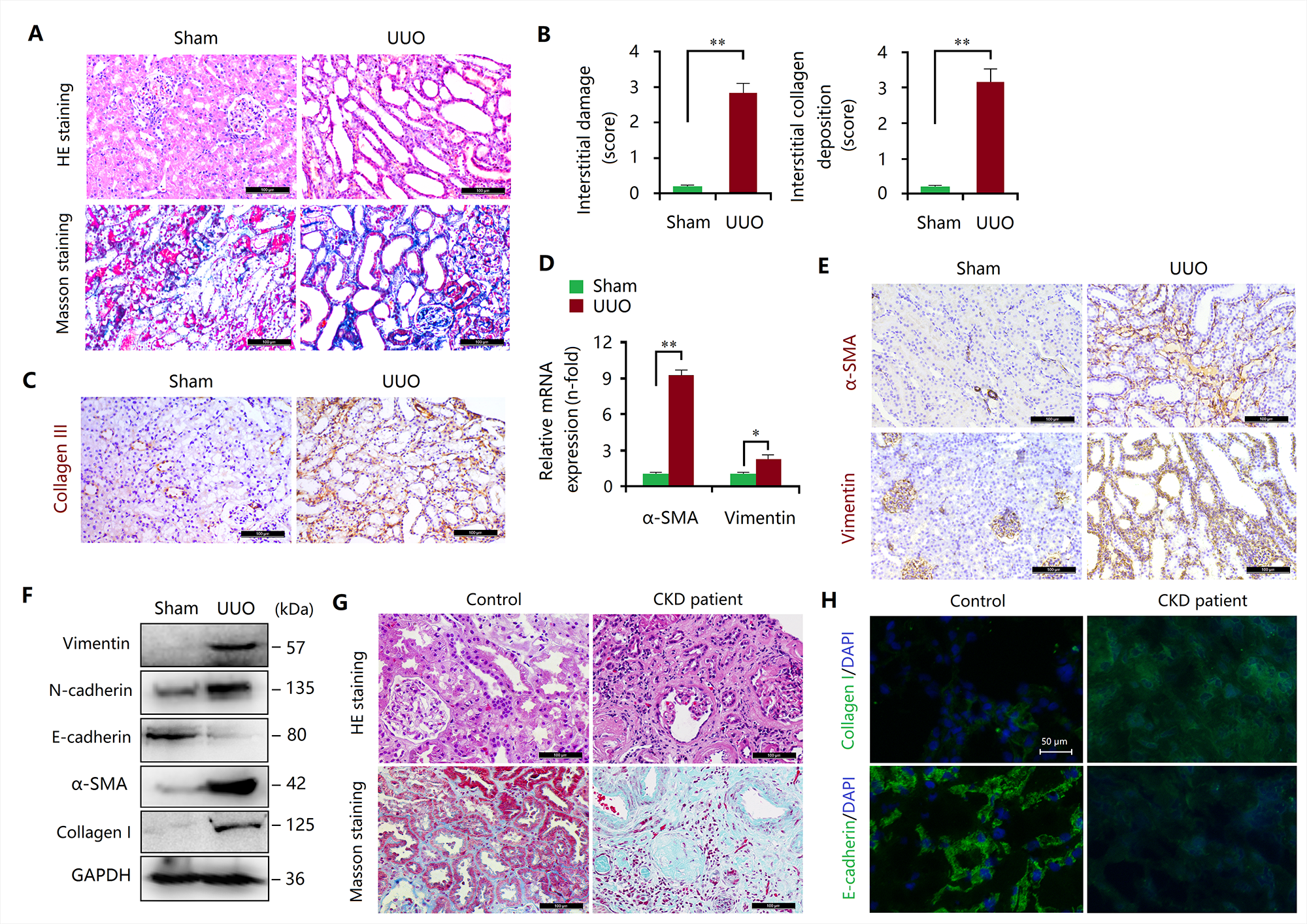

Supplement: Supplementary file 1 — Additional file 1: Figure S1. Myofibroblastic phenotype and fibrosis in UUO kidneys. (A) HE staining showed obvious kidney injury in UUO kidneys, and Masson’s trichrome staining revealed excessive deposition of total collagen. Bar = 100 μm. (B) Ureteral obstruction induced marked increases in interstitial damage and interstitial collagen deposition in kidneys of rats. (C) Increased expression of collagen III in UUO kidneys compared with the sham group. Bar = 100 μm. (D) Increased mRNA expression levels of α-SMA and vimentin in UUO kidneys compared with the sham group. (E) Increased expression of vimentin in UUO kidneys compared with the sham group. Bar = 100 μm. (F) Increased protein expression of vimentin, N-cadherin, α-SMA, and collagen I, and decreased expression of E-cadherin in UUO kidneys compared with the sham group. (G) HE and Masson’s trichrome staining showed obvious tubular and interstitial damage and excessive collagen deposition in CKD patients. Bar = 100 μm. (H) Increased expression of collagen I and decreased expression of E-cadherin in CKD patients. Bar = 50 μm. All data are presented as means ± SDs. *P < 0.05, **P < 0.01 versus the sham group. [file 12964_2019_455_MOESM1_ESM.tif]

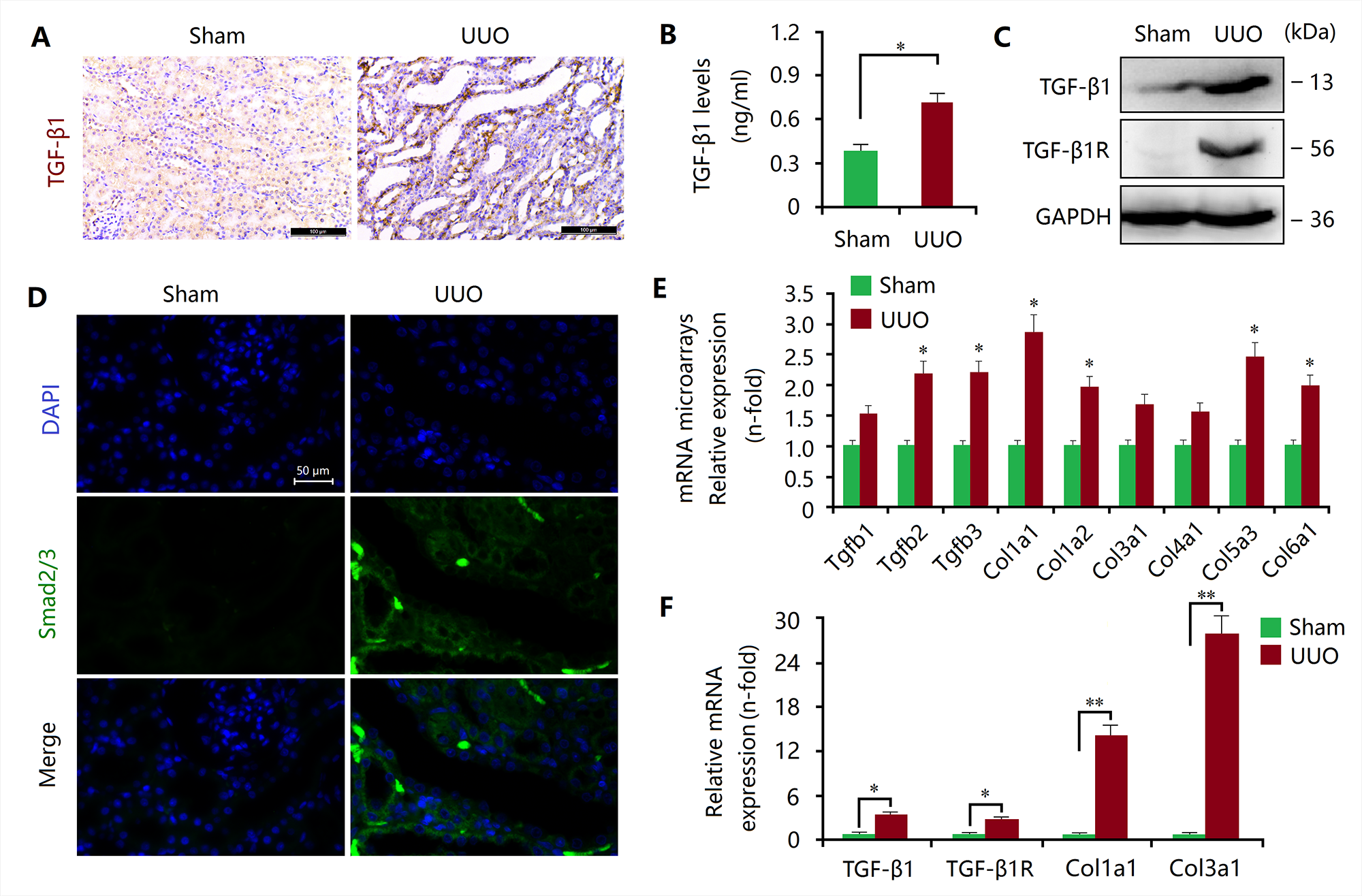

Supplement: Supplementary file 2 — Additional file 2: Figure S2. TGF-β1/Smad2/3 signalling is activated during fibrosis in UUO kidneys. (A) The expression of TGF-β1 in UUO kidneys was significantly increased. Bar = 100 μm. (B) ELISA assay revealed enhanced levels of TGF-β1 in UUO rats. (C) The expression levels of TGF-β1 and TGF-β1R in UUO kidneys were significantly increased. (D) The expression and location of Smad2/3 in UUO rats. Bar = 50 μm. (E) mRNA microarrays revealed that the expression levels of TGF-β1- and ECM-associated genes were enhanced in UUO rats. (F) The mRNA expression levels of TGF-β1, TGF-β1R, Col1α1, and Col3α1 were increased in UUO kidneys compared with those in the sham. All data are presented as means ± SDs. *P < 0.05, **P < 0.01 versus the sham group. [file 12964_2019_455_MOESM2_ESM.tif]

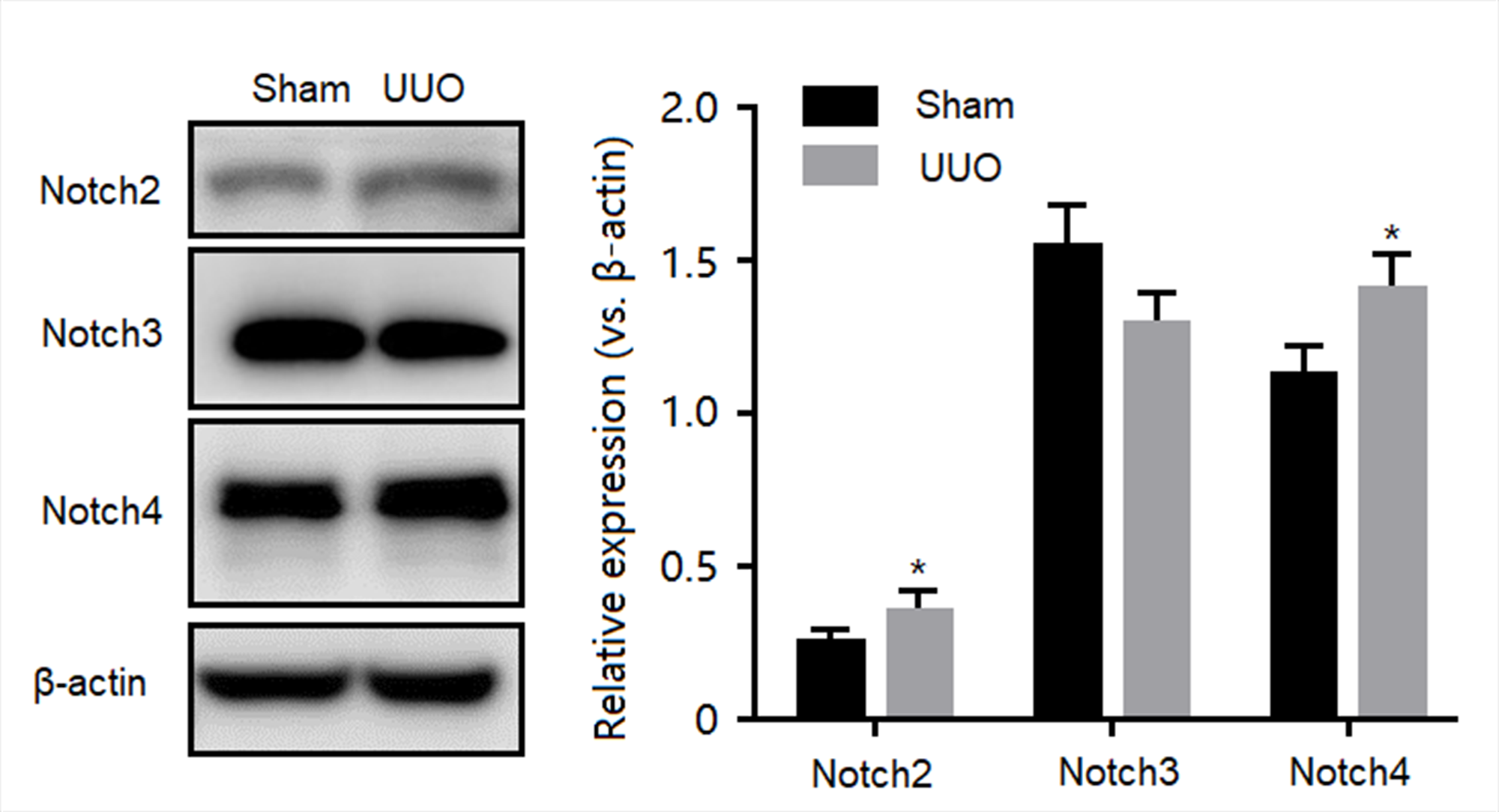

Supplement: Supplementary file 3 — Additional file 3: Figure S3. The expression of Notch2, Notch3, and Notch4 in renal fibrosis. Increased protein expression of Notch2 and Notch4 in UUO kidneys compared with the sham group. However, the expression of Notch3 did not show any difference. All data are presented as means ± SDs. *P < 0.05 versus the sham group. [file 12964_2019_455_MOESM3_ESM.tif]

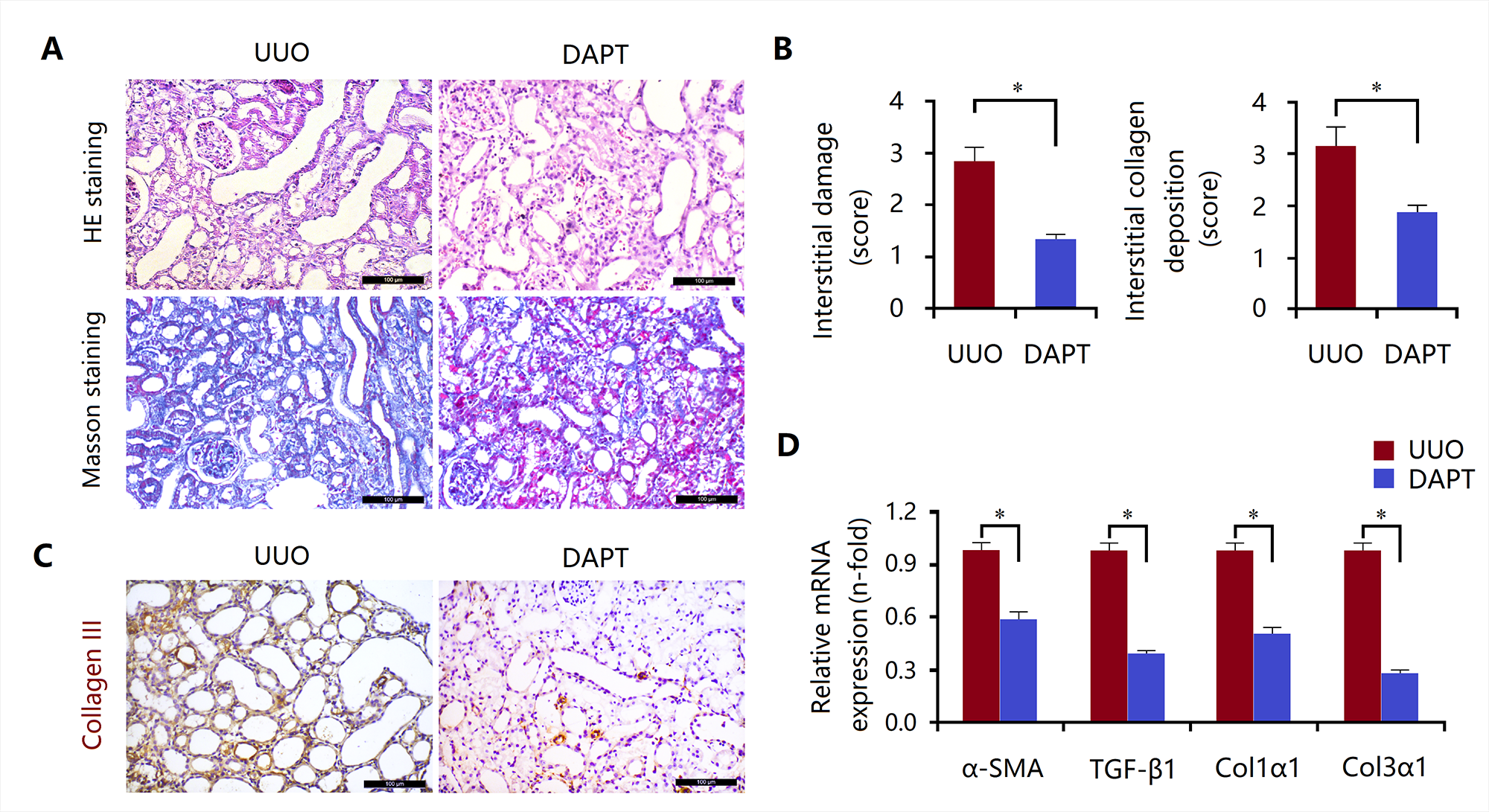

Supplement: Supplementary file 4 — Additional file 4: Figure S4. Inhibition of Notch1 signalling with DAPT reduces fibrosis. (A) Kidney injury and excessive deposition of total collagen in UUO kidneys were reduced by DAPT administration. Bar = 100 μm. (B) The interstitial damage and interstitial collagen deposition in UUO kidneys were evaluated according to HE and Masson’s trichrome staining. (C) Increased expression of type III collagen in UUO kidneys was inhibited by DAPT treatment. Bar = 100 μm. (D) qRT-PCR showed that increased mRNA expression levels of α-SMA, TGF-β1, Col1α1, and Col3α1 in UUO kidneys were inhibited by DAPT treatment. All data are presented as means ± SDs. *P < 0.05 versus the control group; #P < 0.05 versus the AA-treated group. [file 12964_2019_455_MOESM4_ESM.tif]

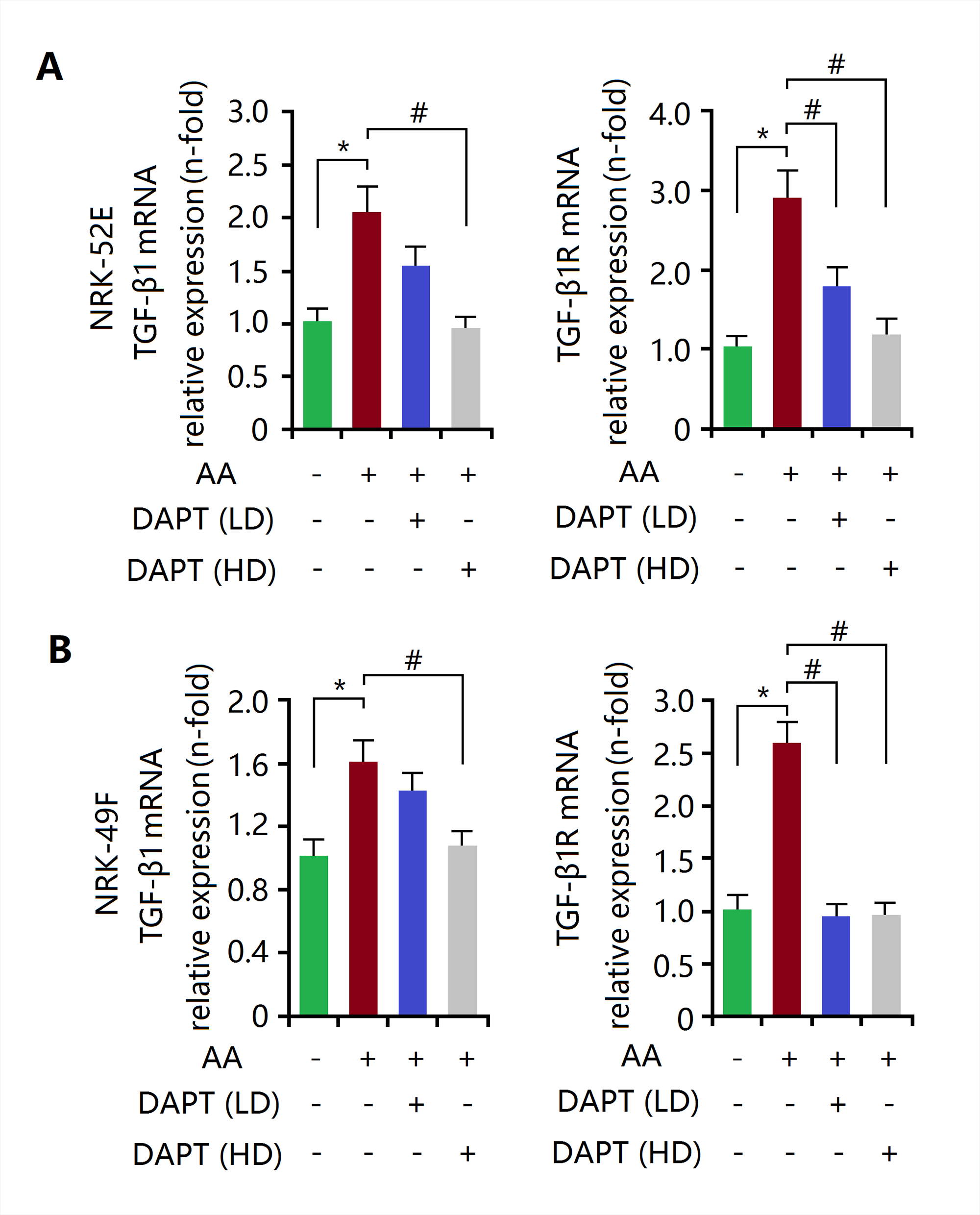

Supplement: Supplementary file 5 — Additional file 5: Figure S5. The TGF-β1 signalling activity in TECs and fibroblasts following AA injury. (A) qRT-PCR showed that increased mRNA expression levels of TGF-β1 and TGF-β1R in AA-treated NRK-52E cells were inhibited by DAPT treatment. (B) qRT-PCR showed that increased mRNA expression levels of TGF-β1 and TGF-β1R in AA-treated NRK-49F cells were inhibited by DAPT treatment. AA, 10 ng/ml; DAPT (HD), high-dose (10 μmol/L); DAPT (LD), low-dose (1 μmol/L). All data are presented as means ± SDs. *P < 0.05 versus the control group; #P < 0.05 versus the AA-treated group. [file 12964_2019_455_MOESM5_ESM.tif]

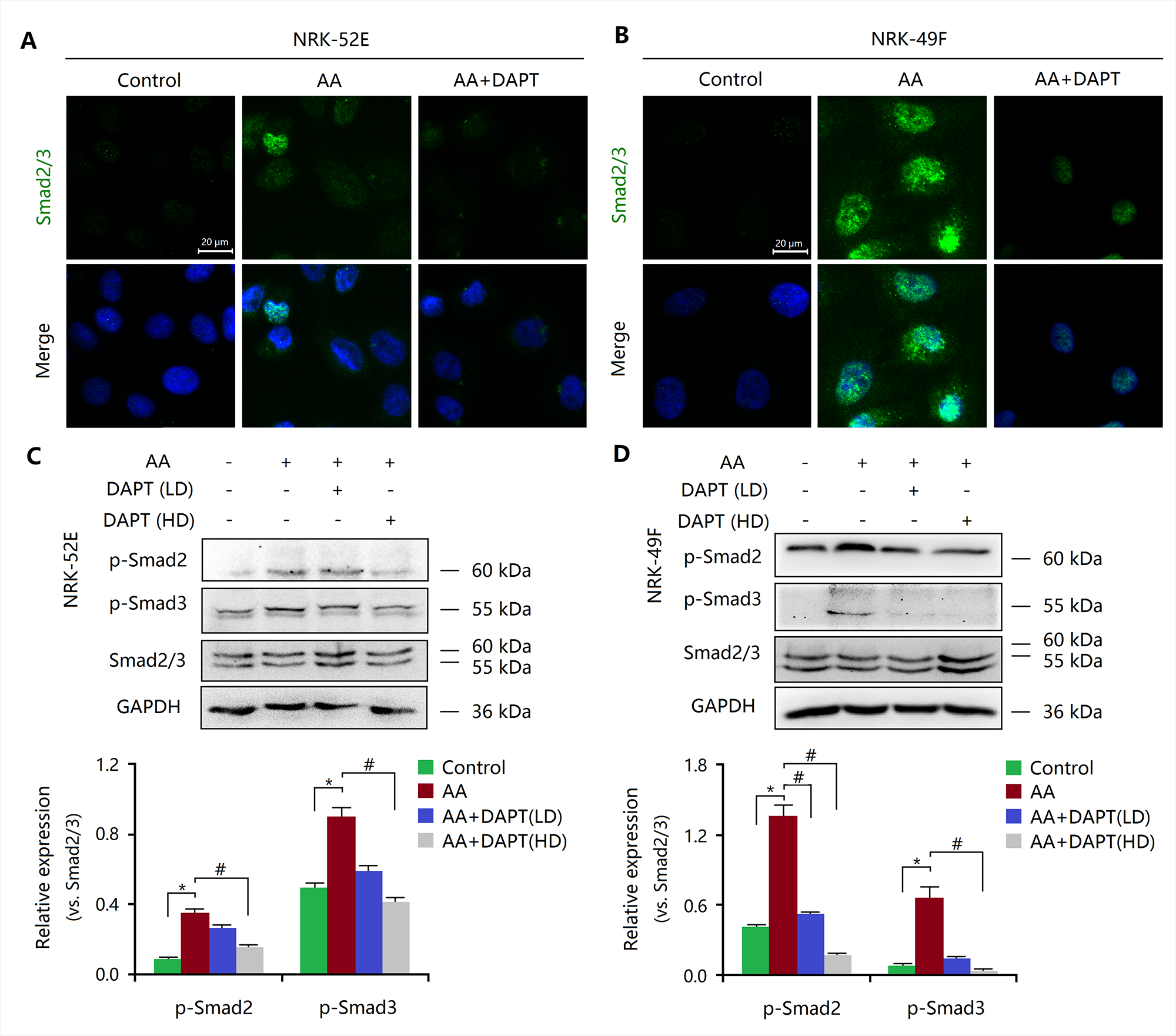

Supplement: Supplementary file 6 — Additional file 6: Figure S6. The Smad2/3 signalling activity in TECs and fibroblasts following AA injury. (A) Immunofluorescence staining indicated upregulated expression and nuclear localization of Smad2/3 in NRK-52E cells after AA treatment, but these effects were inhibited by DAPT. Bar = 20 μm. (B) Immunofluorescence staining indicated upregulated expression and nuclear location of Smad2/3 in NRK-49F cells after AA treatment, but it was inhibited by DAPT. Bar = 20 μm. (C) Upregulated phosphorylation levels of Smad2 and Smad3 in AA-treated NRK-52E cells were inhibited by DAPT. (D) Upregulated phosphorylation levels of Smad2 and Smad3 in AA-treated NRK-49F cells were inhibited by DAPT. AA, 10 ng/ml; DAPT (HD), high-dose (10 μmol/L); DAPT (LD), low-dose (1 μmol/L). All data are presented as means ± SDs. *P < 0.05 versus the control group; #P < 0.05 versus the AA-treated group. [file 12964_2019_455_MOESM6_ESM.tif]

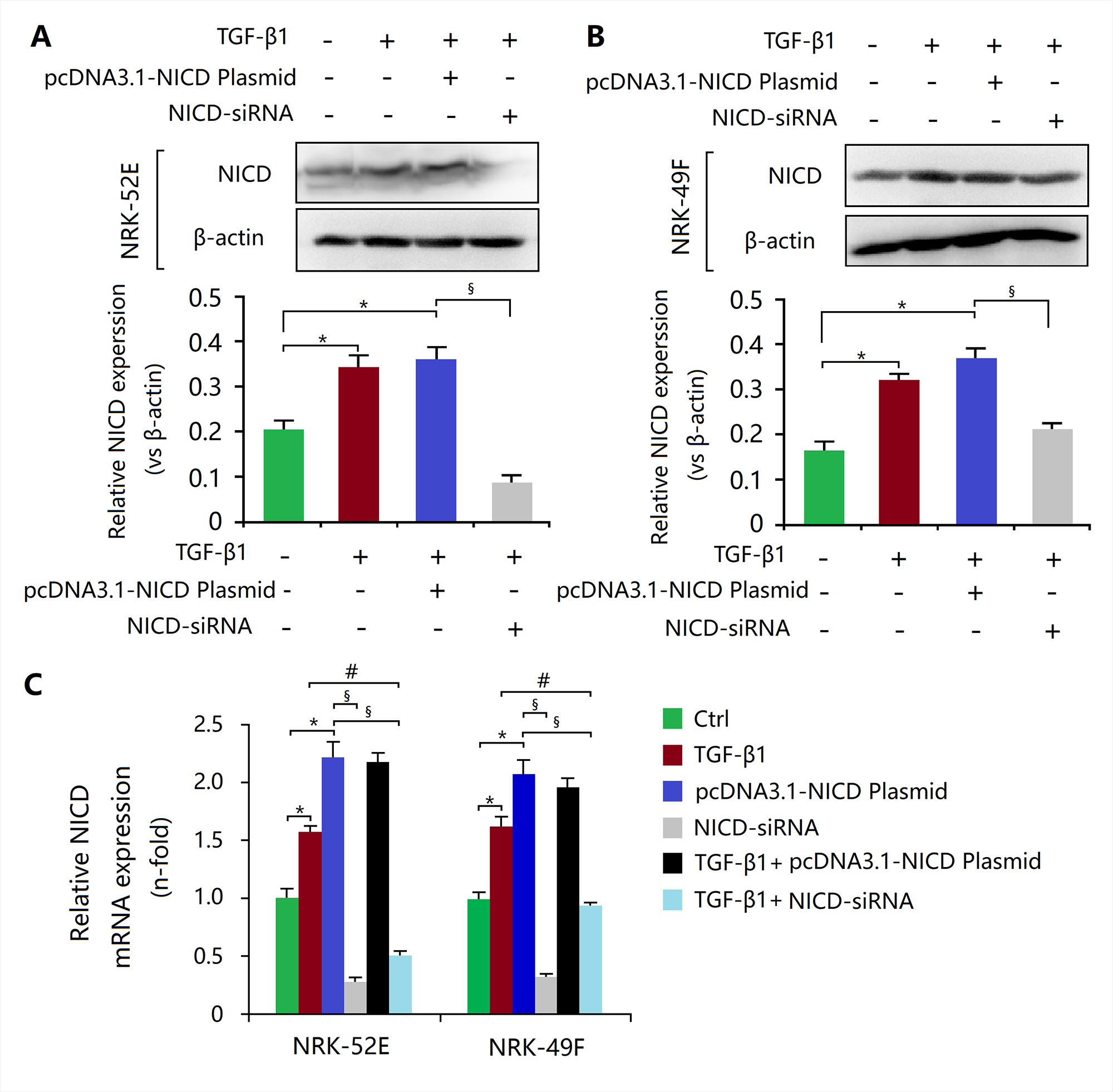

Supplement: Supplementary file 7 — Additional file 7: Figure S7. The NICD activity in TECs and fibroblasts treated with TGF-β1, pcDNA3.1-NICD plasmid, or NICD siRNA. (A) The expression of NICD in NRK-52E cells treated with TGF-β1, pcDNA3.1-NICD plasmid, or NICD siRNA. (B) The expression of NICD in NRK-49F cells treated with TGF-β1, pcDNA3.1-NICD plasmid, or NICD siRNA. (C) The mRNA expression of NICD in NRK-52E and NRK-49F cells treated with TGF-β1, pcDNA3.1-NICD plasmid, or NICD siRNA. All data are presented as means ± SDs. TGF-β1, 5 ng/ml. *P < 0.05, versus the control group; #P < 0.05 versus the TGF-β1-treated group; §P < 0.05 versus the pcDNA3.1-NICD plasmid-treated group. [file 12964_2019_455_MOESM7_ESM.tif]
