# Supplementary material for: Epithelial and interstitial Notch1 activity contributes to the myofibroblastic phenotype and fibrosis
Source: Cell Commun Signal. 2019 Nov 12;17:145. doi: 10.1186/s12964-019-0455-y (PMC6849313; doi:10.1186/s12964-019-0455-y)
Supplement: Supplementary file 8 — Additional file 8: Table S1. Two-step real-time RT-PCR primers for analysis. [file 12964_2019_455_MOESM8_ESM.docx]

**Table S1** Two-step real-time RT-PCR primers for analysis

| Gene | Forward sequence (5’→3’) | Reverse sequence (5’→3’) | NCBI accession | Product size |
| --- | --- | --- | --- | --- |
| α-SMA | GGCATCCACGAAACCACCT | CCGCCGATCCAGACAGAAT | NM_031004.2 | 212 bp |
| Bmp7 | GTGGTCAACCCTCGGCACA | GGCGTCTTGGAGCGATTCTG | NM_001191856.1 | 215bp |
| c-Myc | CTTCCCCTACCCGCTCAACGAC | CACATCAATTTCTTCCTCATCA | NM_012603.2 | 208 bp |
| Col1α1 | GATCCTGCCGATGTCGCTAT | GGAGGTCTTGGTGGTTTTGTATTC | NM_053304.1 | 276 bp |
| Col3α1 | AAGGCTGAAGGAAATAGC | AATGTCATAGGGTGCGATA | NM_032085.1 | 147 bp |
| E-cadherin | GTGCCACCACCAAAGATA | GGCTGAGACAACCCTAAT | NM_031334.1 | 195 bp |
| Notch1 | CTGCCTTCGTGCTCCTGTTCTTT | GAGGGGTTCTCTCCGCTTCTTCT | NM_001105721.1 | 137 bp |
| Numb | GACACGGGGGAAAGACTGAG | TTCCCGCTTCTGTTTACGCT | NM_133287.1 | 75 bp |
| Jagged1 | CTGCTTGAATGGGGGTCACT | CACGATTGTAGCATTGGGCG | NM_019147.1 | 146 bp |
| Smad2 | GCCGCCCGAAGGGTAGAT | TTCTGTTCTCCACCACCTGC | NM_001277450.1 | 164 bp |
| Smad3 | TTTACTGGACTGAGGTTGGCTG | GTTTGGAGAGCCTGCGTCCAT | XM_008766216.2 | 195 bp |
| TGF-β1 | CTGCTGACCCCCACTGATAC | AGCCCTGTATTCCGTCTCCT | NM_021578.2 | 94 bp |
| TGF-β1R | ACTCCCAACTACAGAAAAGCA | ACTCCCAACTACAGAAAAGCA | NM_012775.2 | 132 bp |
| Tp53 | TCACCATCATCACACTGGAAGACTC | TTGGGCAGTGCTCGCTTAGT | NM_000546.5 | 175 bp |
| vimentin | TGACCGCTTCGCCAACTAC | CGCAACTCCCTCATCTCCTC | NM_031140.1 | 141 bp |
| Hes1 | CAACACGACACCGGACAAAC | CGGAGGTGCTTCACTGTCAT | NM_024360.3 | 204 bp |
| Hes6 | AGGGGTTTGCACTAAGGCG | CCTGTGGGAAACGGCTACTGA | NM_001013179.1 | 207 bp |
| Hey1 | CTGTCGCCGCCATGCTTCTC | CCTGTGAGGTGTCAAGACTCGTAATG | NM_001191845.1 | 81 bp |
| Hey2 | TTTCCCATGCTCCCATCCAATGC | TTGTTCCACTGCTTGTCTGCTGAG | NM_130417.1 | 118 bp |
| β-actin | GAGAGGGAAATCGTGCGTGAC | CCATACCCAGGAAGGAAGGCT | NM_031144.3 | 196 bp |
