# Supplementary material for: Epithelial and interstitial Notch1 activity contributes to the myofibroblastic phenotype and fibrosis
Source: Cell Commun Signal. 2019 Nov 12;17:145. doi: 10.1186/s12964-019-0455-y (PMC6849313; doi:10.1186/s12964-019-0455-y)
Supplement: Supplementary file 9 — Additional file 9: Table S2. Primers for NICD siRNA and overexpression. [file 12964_2019_455_MOESM9_ESM.docx]

**Table S2** Primers for NICD siRNA and overexpression

|  | Gene | Sequence |
| --- | --- | --- |
| NICD siRNA | NRK-49F | GGACAAACUGUGACAUCAAdTdT |
|  | NRK-52E | GCGUGUGCACAGAAGGUUAdTdT |
| NICD overexpression | NICD-F | CG***GGATCC***ATGGTGCTGCTGTCCCGCAAG  BamHI |
|  | NICD-R | CG***GAATTC***CTTAAATGCCTCTGGAATGTG  EcoRI |
